# Interpretability of Multivariate Brain Maps in Linear Brain Decoding: Definition, and Heuristic Quantification in Multivariate Analysis of MEG Time-Locked Effects

**DOI:** 10.3389/fnins.2016.00619

**Published:** 2017-01-23

**Authors:** Seyed Mostafa Kia, Sandro Vega Pons, Nathan Weisz, Andrea Passerini

**Affiliations:** ^1^Department of Information Engineering and Computer Science, University of TrentoTrento, Italy; ^2^Fondazione Bruno KesslerTrento, Italy; ^3^Pattern Analysis and Computer Vision, Istituto Italiano di TecnologiaGenova, Italy; ^4^Division of Physiological Psychology, Centre for Cognitive Neuroscience, University of SalzburgSalzburg, Austria

**Keywords:** brain decoding, brain mapping, interpretation, model selection, MEG

## Abstract

Brain decoding is a popular multivariate approach for hypothesis testing in neuroimaging. Linear classifiers are widely employed in the brain decoding paradigm to discriminate among experimental conditions. Then, the derived linear weights are visualized in the form of multivariate brain maps to further study spatio-temporal patterns of underlying neural activities. It is well known that the brain maps derived from weights of linear classifiers are hard to interpret because of high correlations between predictors, low signal to noise ratios, and the high dimensionality of neuroimaging data. Therefore, improving the interpretability of brain decoding approaches is of primary interest in many neuroimaging studies. Despite extensive studies of this type, at present, there is no formal definition for interpretability of multivariate brain maps. As a consequence, there is no quantitative measure for evaluating the interpretability of different brain decoding methods. In this paper, first, we present a theoretical definition of interpretability in brain decoding; we show that the interpretability of multivariate brain maps can be decomposed into their reproducibility and representativeness. Second, as an application of the proposed definition, we exemplify a heuristic for approximating the interpretability in multivariate analysis of evoked magnetoencephalography (MEG) responses. Third, we propose to combine the approximated interpretability and the generalization performance of the brain decoding into a new multi-objective criterion for model selection. Our results, for the simulated and real MEG data, show that optimizing the hyper-parameters of the regularized linear classifier based on the proposed criterion results in more informative multivariate brain maps. More importantly, the presented definition provides the theoretical background for quantitative evaluation of interpretability, and hence, facilitates the development of more effective brain decoding algorithms in the future.

## 1. Introduction

Understanding the mechanisms of the brain has been a crucial topic throughout the history of science. Ancient Greek philosophers envisaged different functionalities for the brain ranging from cooling the body to acting as the seat of the rational soul and the center of sensation (Crivellato and Ribatti, 2007). Modern cognitive science, emerging in the twentieth century, provides better insight into the brain's functionality. In cognitive science, researchers usually analyze recorded brain activity and behavioral parameters to discover the answers of *where, when*, and *how* a brain region participates in a particular cognitive process.

To answer the key questions in cognitive science, scientists often employ mass-univariate hypothesis testing methods to test scientific hypotheses on a large set of independent variables (Groppe et al., 2011a; Maris, 2012). Mass-univariate hypothesis testing is based on performing multiple tests, e.g., *t*-tests, one for each unit of the neuroimaging data, i.e., independent variables. The high spatial and temporal granularity of the univariate tests provides fair level of interpretability. On the down side, the high dimensionality of neuroimaging data requires a large number of tests that reduces the sensitivity of these methods after multiple comparison correction (Bzdok et al., 2016). Although techniques such as the non-parametric cluster-based permutation test (Bullmore et al., 1996; Maris and Oostenveld, 2007), by weak rather strong control of family-wise error rate, offer more sensitivity, they still experience low sensitivity to brain activities that are narrowly distributed in time and space (Groppe et al., 2011a,b). The multivariate counterpart of mass-univariate analysis, known generally as multivariate pattern analysis, have the potential to overcome these deficits. Multivariate approaches are capable of identifying complex spatio-temporal interactions between different brain areas with higher sensitivity and specificity than univariate analysis (van Gerven et al., 2009), especially in group analysis of neuroimaging data (Davis et al., 2014).

*Brain decoding* (Haynes and Rees, 2006) is a multivariate technique that delivers a model to predict the mental state of a human subject based on the recorded brain signal. There are two potential applications for brain decoding: (1) brain-computer interfaces (BCIs) (Wolpaw et al., 2002), and (2) multivariate hypothesis testing (Bzdok et al., 2016). In the first case, a brain decoder with maximum prediction power is desired. In the second case, in addition to the prediction power, extra information on the spatio-temporal nature of a cognitive process is desired. In this study, we are interested in the second application of brain decoding that can be considered a multivariate alternative for mass-univariate hypothesis testing. Further, we mainly focus on the linear brain decoding because of its wider usage in analyzing inherently small sample size and high dimensional neuroimaging data, compared to the complex (Cox and Savoy, 2003; LaConte et al., 2005) and non-transparent (Lipton et al., 2016) non-linear models.

In linear brain decoding, linear classifiers are used to assess the relation between independent variables, i.e., features, and dependent variables, i.e., cognitive tasks (Besserve et al., 2007; Pereira et al., 2009; Lemm et al., 2011). This assessment is performed by solving an optimization problem that assigns weights to each independent variable. Currently, brain decoding is the gold standard in multivariate analysis for functional magnetic resonance imaging (fMRI) (Haxby et al., 2001; Cox and Savoy, 2003; Mitchell et al., 2004; Norman et al., 2006) and magnetoencephalogram/electroencephalogram (MEEG) studies (Parra et al., 2003; Rieger et al., 2008; Carroll et al., 2009; Chan et al., 2011; Huttunen et al., 2013; Vidaurre et al., 2013; Abadi et al., 2015). It has been shown that brain decoding can be used in combination with brain encoding (Naselaris et al., 2011) to infer the causal relationship between stimuli and responses (Weichwald et al., 2015).

In *Brain mapping* (Kriegeskorte et al., 2006) the pre-computed quantities, e.g., univariate statistics or weights of a linear classifier, are assigned to the spatio-temporal representation of neuroimaging data in order to reveal functionally specialized brain regions which are activated by a certain cognitive task. In its multivariate form, brain mapping uses the learned parameters from brain decoding to produce brain maps, in which the engagement of different brain areas in a cognitive task is visualized. Intuitively, the interpretability of a brain decoder refers to the level of information that can be reliably derived by an expert from the resulting maps. From the cognitive neuroscience perspective, a brain map is considered *interpretable* if it enables a scientist to find out the answers of three key questions: “*where, when*, and *how* does a brain region contribute to a cognitive function?”

In fact, a classifier only answers the question of *what* is the most likely label of a given unseen sample (Baehrens et al., 2010). This fact is generally known as knowledge extraction gap (Vellido et al., 2012) in the machine learning context. Thus far, many efforts have been devoted to filling the knowledge extraction gap of linear and non-linear data modeling methods in different areas such as computer vision (Bach et al., 2015), signal processing (Montavon et al., 2013), chemometrics (Yu et al., 2015), bioinformatics (Hansen et al., 2011), and neuroinformatics (Haufe et al., 2013). In the context of neuroimaging, the knowledge extraction gap in classification is generally known as the interpretation problem (Sabuncu, 2014; Haynes, 2015; Naselaris and Kay, 2015). Therefore, improving the interpretability of linear brain decoding and associated brain maps is a primary goal in the brain imaging literature (Strother et al., 2014). The lack of interpretability of multivariate brain maps is a direct consequence of low signal-to-noise ratios (SNRs), high dimensionality of whole-scalp recordings, high correlations among different dimensions of data, and cross-subject variability (Besserve et al., 2007; Anderson et al., 2011; Blankertz et al., 2011; Brodersen et al., 2011; Langs et al., 2011; Lemm et al., 2011; Varoquaux et al., 2012; Kauppi et al., 2013; Haufe et al., 2014a; Olivetti et al., 2014; Taulu et al., 2014; Varoquaux and Thirion, 2014; Haynes, 2015; Wang et al., 2015). At present, two main approaches are proposed to enhance the interpretability of multivariate brain maps: (1) introducing new metrics into the model selection procedure, and (2) introducing new hybrid penalty terms for regularization.

The first approach to improving the interpretability of brain decoding concentrates on the model selection procedure. Model selection is a procedure in which the best values for the hyper-parameters of a model are determined (Lemm et al., 2011). The selection process is generally performed by considering the generalization performance, i.e., the accuracy, of a model as the decisive criterion. Rasmussen et al. (2012) showed that there is a trade-off between the spatial reproducibility and the prediction accuracy of a classifier; therefore, the reliability of maps cannot be assessed merely by focusing on their prediction accuracy. To utilize this finding, they incorporated the spatial reproducibility of brain maps in the model selection procedure. An analogous approach, using a different definition of spatial reproducibility, is proposed by Conroy et al. (2013). Beside spatial reproducibility, the stability of the classifiers (Bousquet and Elisseeff, 2002) is another criterion that is used in combination with generalization performance to enhance the interpretability. For example Yu (2013) and Lim and Yu (2016) showed that incorporating the stability of models into cross-validation improves the interpretability of the estimated parameters (by linear models).

The second approach to improving the interpretability of brain decoding focuses on the underlying mechanism of regularization. The main idea behind this approach is two-fold: 1) customizing the regularization terms to address the ill-posed nature of brain decoding problems (where the number of samples is much less than the number of features; Mørch et al., 1997; Varoquaux and Thirion, 2014), and (2) combining the structural and functional prior knowledge with the decoding process so as to enhance the neurophysiological plausibility of the models. Group Lasso (Yuan and Lin, 2006) and total-variation penalty (Tibshirani et al., 2005) are two effective methods using this technique (Rish et al., 2014; Xing et al., 2014). Sparse penalized discriminant analysis (Grosenick et al., 2008), group-wise regularization (van Gerven et al., 2009), smoothed-sparse logistic regression (de Brecht and Yamagishi, 2012), total-variation ℓ_1_ penalization (Michel et al., 2011; Gramfort et al., 2013), the graph-constrained elastic-net (Grosenick et al., 2009, 2013), and social-sparsity (Varoquaux et al., 2016) are examples of brain decoding methods in which regularization techniques are employed to improve the interpretability of linear brain decoding models.

Recently, taking a new approach to the problem, Haufe et al. questioned the interpretability of weights of linear classifiers because of the contribution of noise in the decoding process (Bießmann et al., 2012; Haufe et al., 2013, 2014b). To address this problem, they proposed a procedure to convert the linear brain decoding models into their equivalent generative models. Their experiments on the simulated and fMRI/EEG data illustrate that, whereas the direct interpretation of classifier weights may cause severe misunderstanding regarding the actual underlying effect, their proposed transformation effectively provides interpretable maps. Despite the theoretical soundness, the intricate challenge of estimating the empirical covariance matrix of the small sample size neuroimaging data (Blankertz et al., 2011) limits the practical application of this method.

In spite of the aforementioned efforts to improve the interpretability of brain decoding, there is still no formal definition for the interpretability of brain decoding in the literature. Therefore, the interpretability of different brain decoding methods are evaluated either qualitatively or indirectly (i.e., by means of an intermediate property). In qualitative evaluation, to show the superiority of one decoding method over the other (or a univariate map), the corresponding brain maps are compared visually in terms of smoothness, sparseness, and coherency using already known facts (see for example, Varoquaux et al., 2012). In the second approach, important factors in interpretability such as spatio-temporal reproducibility are evaluated to indirectly assess the interpretability of results (see for example, Langs et al., 2011; Rasmussen et al., 2012; Conroy et al., 2013; Kia et al., 2016). Despite partial effectiveness, there is no general consensus regarding the quantification of these intermediate criteria. For example, in the case of spatial reproducibility, different methods such as correlation (Rasmussen et al., 2012; Kia et al., 2016), dice score (Langs et al., 2011), or parameter variability (Conroy et al., 2013; Haufe et al., 2013) are used for quantifying the stability of brain maps, each of which considers different aspects of local or global reproducibility.

With the aim of filling this gap, our contribution is three-fold: (1) Assuming that the true solution of brain decoding is available, we present a theoretical definition of the interpretability. The presented definition is simply based on cosine proximity in the parameter space. Furthermore, we show that the interpretability can be decomposed into the reproducibility and the representativeness of brain maps. (2) As a proof of the concept, we exemplify a practical heuristic based on event-related fields for quantifying the interpretability of brain maps in time-locked analysis of MEG data. (3) Finally, we propose the combination of the interpretability and the performance of the brain decoding as a new Pareto optimal multi-objective criterion for model selection. We experimentally, on both simulated and real data, show that incorporating the interpretability into the model selection procedure provides more reproducible, more neurophysiologically plausible, and (as a result) more interpretable maps. Furthermore, in comparison with a standard univariate analysis, we show the proposed paradigm offers more sensitivity while preserving the interpretability of results.

## 2. Materials and methods

### 2.1. Notation and background

Let $X\in {\mathbb{R}}^{p}$ be a manifold in Euclidean space that represents the input space and Y∈ℝ be the output space, where $Y=\Phi^{*}\left(X\right)$. Then, let *S* = {**Z** = (**X**, **Y**) | *z*_1_ = (*x*_1_, *y*_1_), …, *z*_*n*_ = (*x*_*n*_, *y*_*n*_)} be a training set of *n* independently and identically distributed (i.i.d) samples drawn from the joint distribution of Z=X×Y based on an unknown Borel probability measure ρ. In the neuroimaging context, **X** indicates the trials of brain recording, e.g., fMRI, MEG, or EEG signals, **Y** represents the experimental conditions or dependent variables, and we have Φ_*S*_ : **X** → **Y** (note the difference between Φ_*S*_ and Φ^*^). The goal of brain decoding is to find the function $\hat{\Phi }:\text{X}\to \text{Y}$ as an estimation of Φ_*S*_. Here on we refer to $\hat{\Phi }$ as a brain decoding model.

As is a common assumption in the neuroimaging context, we assume the true solution of a brain decoding problem is among the family of linear functions H ($\Phi^{*}\in H$). Therefore, the aim of brain decoding reduces to finding an empirical approximation of Φ_*S*_, indicated by $\hat{\Phi }$, among all Φ∈H. This approximation can be obtained by estimating the predictive conditional density ρ(**Y** | **X**) by training a parametric model ρ(**Y** | **X**, Θ) (i.e., a likelihood function), where Θ denotes the parameters of the model. Alternatively, Θ can be estimated by solving a risk minimization problem:

(1) $$\begin{array}{l} \begin{array}{c} \hat{\Theta }=\underset{\Theta }{\mathrm{argmin}}L\left(\text{X}\Theta ,\text{Y}\right)+\lambda \Omega \left(\Theta \right) \end{array} \end{array}$$

where $\hat{\Theta }$ is the parameter of $\hat{\Phi }$, $L:\text{Z}\times \text{Z}\to {\mathbb{R}}^{+}$ is the loss function, Ω : ℝ^*p*^ → ℝ^+^ is the regularization term, and λ is a hyper-parameter that controls the amount of regularization. There are various choices for Ω, each of which reduces the hypothesis space H to $H^{\prime }\subset H$ by enforcing different prior functional or structural constraints on the parameters of the linear decoding model (see for example, Tibshirani, 1996b; Tibshirani et al., 2005; Zou and Hastie, 2005; Jenatton et al., 2011). The amount of regularization λ is generally decided using cross-validation or other data perturbation methods in the model selection procedure.

In the neuroimaging context, the estimated parameters of a linear decoding model $\hat{\Theta }$ can be used in the form of a brain map so as to visualize the discriminative neurophysiological effect. Although the magnitude of $\hat{\Theta }$ (i.e., the 2-norm of $\hat{\Theta }$) is affected by the dynamic range of data and the level of regularization, it has no effect on the predictive power and the interpretability of maps. On the other hand, the direction of $\hat{\Theta }$ affects the predictive power and contains information regarding the importance of and relations among predictors. This type of relational information is very useful when interpreting brain maps in which the relation between different spatio-temporal independent variables can be used to describe how different brain regions interact over time for a certain cognitive process. Therefore, we refer to the normalized parameter vector of a linear brain decoder in the unit hyper-sphere as a multivariate brain map (MBM); we denote it by $\vec{\Theta }$ where $\vec{\Theta }={\frac{\Theta }{\left||\Theta |\right|}}_{2}$ (||.||_2_ represents the 2-norm of a vector).

As shown in Equation (1), learning occurs using the sampled data. In other words, in the learning paradigm, we attempt to minimize the loss function with respect to Φ_*S*_ (and not Φ^*^) (Cucker and Smale, 2002). Therefore, all of the implicit assumptions (such as linearity) regarding Φ^*^ might not hold on Φ_*S*_, and vice versa. The *irreducible error* ε is the direct consequence of sampling; it sets a lower bound on the error, where we have:

(2) $$\begin{array}{l} \Phi_{S}\left(\text{X}\right)=\Phi^{*}\left(\text{X}\right)+\varepsilon \end{array}$$

The distribution of ε dictates the type of loss function L in Equation (1). For example, assuming a Gaussian distribution with mean 0 and variance σ^2^ for ε implies the least squares loss function (Wu et al., 2006).

### 2.2. Interpretability of multivariate brain maps: theoretical definition

In this section, we present a theoretical definition for the interpretability of linear brain decoding models and their associated MBMs. Consider a linearly separable brain decoding problem in an ideal scenario where ε = 0 and *rank*(**X**) = *p*. In this case, the ideal solution of brain decoding, Φ^*^, is linear and its parameters Θ^*^ are *unique* and neurophysiologically *plausible* (van Ede and Maris, 2016). The unique parameter vector Θ^*^ can be computed as follows:

(3) $$\begin{array}{l} \Theta^{*}=\Sigma_{\text{X}}^{-1}{\text{X}}^{T}\text{Y} \end{array}$$

where Σ_**X**_ represents the covariance of **X**. Using Θ^*^ as the reference, we define the *strong-interpretability* of an MBM as follows:

Definition 1. An MBM $\vec{\hat{\Theta }}$ associated with a linear brain decoding model $\hat{\Phi }$ is “strongly-interpretable” if and only if $\vec{\hat{\Theta }}\propto \Theta^{*}$.

It can be shown that, in practice, the estimated solution of a linear brain decoding problem is not strongly-interpretable because of the inherent limitations of neuroimaging data, such as uncertainty (Aggarwal and Yu, 2009) in the input and output space (ε ≠ 0), the high dimensionality of data (*n* ≪ *p*), and the high correlation between predictors (*rank*(**X**) < *p*). With these limitations in mind, even though in practice the solution of linear brain decoding is not strongly-interpretable, one can argue that some are more interpretable than others. For example, in the case in which Θ^*^ ∝ [0, 1]^*T*^, a linear classifier where $\vec{\hat{\Theta }}\propto {\left[0.1,1.2\right]}^{T}$ can be considered more interpretable than a linear classifier where $\vec{\hat{\Theta }}\propto {\left[2,1\right]}^{T}$. This issue raises the following question:

Problem 1. Let *S* be a training set of *n* i.i.d samples drawn from the joint distribution of Z=X×Y, and *P*(*S*) be the probability of drawing a certain *S* from Z. Assume $\vec{\hat{\Theta }}$ is the MBM of a linear brain decoding model $\hat{\Phi }$ on *S* (estimated using Equation 1 for a certain loss function L, regularization term Ω, and hyper-parameter λ). How can we quantify the proximity of $\hat{\Phi }$ to the strongly-intrepretable solution of the brain decoding problem Φ^*^?

To answer this question, considering the uniqueness and the plausibility of Φ^*^ as the two main characteristics that convey its strong-interpretability, we define the interpretability as follows:

Definition 2. Let *S*, *P*(*S*), and $\vec{\hat{\Theta }}$ be as defined in Problem 1. Then, assume α be the angle between $\vec{\hat{\Theta }}$ and ${\vec{\Theta }}^{*}$. The “interpretability” (0 ≤ η_Φ_ ≤ 1) of a linear brain decoding model $\hat{\Phi }$ is defined as follows:
  (4) $$\begin{array}{l} \eta_{\Phi }={𝔼}_{P\left(S\right)}\left[\cos\left(\alpha \right)\right] \end{array}$$

In practice, only a limited number of samples are available. Therefore, perturbation techniques are used to imitate the sampling procedure. Let *S*^1^, …, *S*^*m*^ be *m* perturbed training sets drawn from *S* via a certain perturbation scheme such as jackknife, bootstrapping (Efron, 1992), or cross-validation (Kohavi, 1995). Assume ${\vec{\hat{\Theta }}}^{1},\ldots ,{\vec{\hat{\Theta }}}^{m}$ are *m* MBMs estimated on the corresponding perturbed training sets, and α^*j*^ (*j* = 1, …, *m*) be the angle between ${\vec{\hat{\Theta }}}^{j}$ and ${\vec{\Theta }}^{*}$. Then, the empirical version of Equation (4) can be rewritten as follows:

(5) $$\begin{array}{l} \eta_{\Phi }=\frac{1}{m}\overset{m}{\underset{j=1}{\sum }}\cos\left(\alpha^{j}\right) \end{array}$$

**Figure 1:**
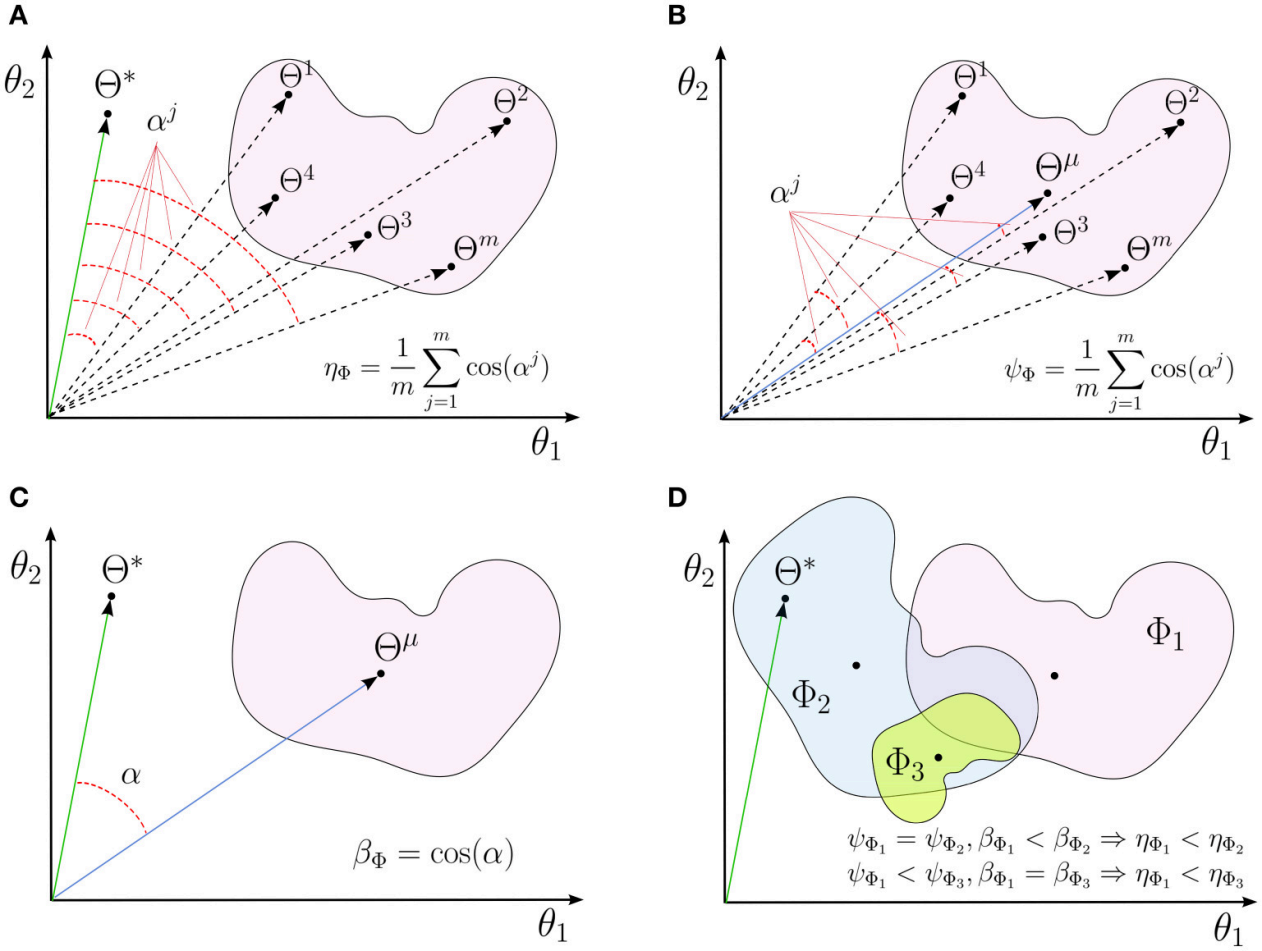
**A schematic illustrations for (A)** interpretability (η_Φ_), **(B)** reproducibility (ψ_Φ_), and **(C)** representativeness (β_Φ_) of a linear decoding model in two dimensions. **(D)** The independent effects of the reproducibility and the representativeness of a model on its interpretability.

Empirically, the interpretability is the mean of cosine similarities between Θ^*^ and MBMs derived fro different samplings of the training set (see Figure 1A for a schematic illustration). In addition to the fact that employing cosine similarity is a common method for measuring the similarity between vectors, we have another strong motivation for this choice. It can be shown that, for large values of *p*, the distribution of the dot product in the unit hyper-sphere, i.e., the cosine similarity, converges to a normal distribution with 0 mean and variance of $\frac{1}{p}$, i.e., $N\left(0,\sqrt{1/p}\right)$. Due to the small variance for a large enough *p* values, any similarity value that is significantly larger than zero represents a meaningful similarity between two high dimensional vectors (see Appendix 6.3 for the mathematical demonstration).

In what follows, we demonstrate how the definition of interpretability is geometrically related to the uniqueness and plausibility characteristics of the true solution of the brain decoding problem.

### 2.3. Interpretability decomposition into reproducibility and representativeness

The trustworthy and informativeness of decoding models are providing two important motivations for improving the interpretability of models (Lipton et al., 2016). The trust of a learning algorithm refers to its ability to converge to a unique solution. On the other hand, the informativeness refers to the level of plausible information that can be derived from a model to assist or advise to a human expert. Therefore, it is expected that the interpretability can be quantified alternatively by assessing its uniqueness and neurophysiological plausibility. In this section, we firstly define the reproducibility and representativeness as measures for quantifying the uniqueness and neurophysiological plausibility of a brain decoding model, respectively. Then we show how these definitions are related to the definition of interpretability.

The high dimensionality and the high correlations between variables are two inherent characteristics of neuroimaging data that negatively affect the uniqueness of the solution of a brain decoding problem. Therefore, a certain configuration of hyper-parameters may result different estimated parameters on different portions of data. Here, we are interested in assessing this variability as a measure for uniqueness. We first define the *main multivariate brain map* as follows:

Definition 3. Let *S*, *P*(*S*), and $\vec{\hat{\Theta }}$ be as defined in Problem 1. The “main multivariate brain map” ${\vec{\Theta }}^{\mu }\in {\mathbb{R}}^{p}$ of a linear brain decoding model $\hat{\Phi }$ is defined as:
  (6) $$\begin{array}{l} {\vec{\Theta }}^{\mu }=\frac{{𝔼}_{P\left(S\right)}\left[\vec{\hat{\Theta }}\right]}{{\left||{𝔼}_{P\left(S\right)}\left[\vec{\hat{\Theta }}\right]|\right|}_{2}} \end{array}$$

Assuming $\theta_{i}^{j}$ be the *i*th (*i* = 1, …, *p*) element of an MBM estimated on the *j*th (*j* = 1, …, *m*) perturbed training set, ${\vec{\Theta }}^{\mu }$ empirically can be estimated by summing up ${\vec{\hat{\Theta }}}^{j}$s (computed on the perturbed training set *S*^*j*^) in the unit hyper-sphere:

(7) $${\vec{\Theta }}^{\mu }=\;\frac{{\left[\sum_{j\;=\;1}^{m}\theta_{1}^{j}\;\sum_{j\;=\;1}^{m}\theta_{2}^{j}\ldots \;\sum_{j\;=\;1}^{m}\theta_{p}^{j}\right]}^{T}}{{\left\|{\left[\sum_{j\;=\;1}^{m}\theta_{1}^{j}\;\sum_{j\;=\;1}^{m}\theta_{2}^{j}\ldots \;\sum_{j\;=\;1}^{m}\theta_{p}^{j}\right]}^{T}\right\|}_{2}}$$

${\vec{\Theta }}^{\mu }$ provides a reference for quantifying the reproducibility of an MBM:

Definition 4. Let *S*, *P*(*S*), and $\vec{\hat{\Theta }}$ be as defined in Problem 1, and ${\vec{\Theta }}^{\mu }$ be the main multivariate brain map of $\hat{\Phi }$. Then, assume α be the angle between ${\vec{\hat{\Theta }}}^{j}$ and ${\vec{\Theta }}^{\mu }$. The “reproducibility” ψ_Φ_ (0 ≤ ψ_Φ_ ≤ 1) of a linear brain decoding model $\hat{\Phi }$ is defined as follows:
  (8) $$\begin{array}{l} \psi_{\Phi }=E_{P\left(S\right)}\left[\cos\left(\alpha \right)\right] \end{array}$$

Let ${\vec{\hat{\Theta }}}^{1},\ldots ,{\vec{\hat{\Theta }}}^{m}$ are *m* MBMs estimated on the corresponding perturbed training sets, and α^*j*^ (*j* = 1, …, *m*) be the angle between ${\vec{\hat{\Theta }}}^{j}$ and ${\vec{\Theta }}^{\mu }$. Then, the empirical version of Eq. 8 can be rewritten as follows:

(9) $$\begin{array}{l} \psi_{\Phi }=\frac{1}{m}\overset{m}{\underset{j=1}{\sum }}\cos\left(\alpha^{j}\right) \end{array}$$

In fact, reproducibility provides a measure for quantifying the dispersion of MBMs, computed over different perturbed training sets, from the main multivariate brain map. Figure 1B shows a schematic illustration for the reproducibility of a linear brain decoding model.

On the other hand, the similarity between the main multivariate brain map of a decoder and the true solution can be employed as a measure for the neurophysiological plausibility of a model. We refer to this similarity as the *representativeness* of a linear brain decoding model:

Definition 5. Let ${\vec{\Theta }}^{\mu }$ be the main multivariate brain map of $\hat{\Phi }$. The “representativeness” β_Φ_ (0 ≤ β_Φ_ ≤ 1) of a linear brain decoding model $\hat{\Phi }$ is defined as the cosine similarity between its main multivariate brain map (${\vec{\Theta }}^{\mu }$) and the parameters of the true solution (${\vec{\Theta }}^{*}$) (see Figure 1C):
  (10) $$\begin{array}{l} \beta_{\Phi }=\frac{\left|{\vec{\Theta }}^{\mu }.{\vec{\Theta }}^{*}\right|}{||{\vec{\Theta }}^{\mu }|{|}_{2}||{\vec{\Theta }}^{*}|{|}_{2}} \end{array}$$

As discussed before, the notion of interpretabilty is tightly related to the uniqueness and plausibility, thus to the reproducibility and representativeness, of a decoding model. The following proposition analytically shows this relationship:

Proposition 1. η_Φ_ = β_Φ_ × ψ_Φ_.

See Appendix 6.1 for a proof. Proposition 1 indicates the interpretability of a linear brain decoding model can be decomposed into its representativeness and reproducibility. Figure 1D illustrates how the reproducibility and the representativeness of a decoding model independently affect its interpretability. Each colored region schematically represents a span of different solutions of the a certain linear model (for example with a certain configuration for its hyper-parameters) on different perturbed training sets. The area of each region schematically visualizes the reproducibility of each model, i.e., the less is the area, the higher is the reproducibility of a model. Further, the angular distance between the centroid of each region (Θ^μ^) and the true solution (Θ^*^) visualizes the representativeness of each corresponding model. While Φ_1_ and Φ_2_ have similar reproducibility, Φ_2_ has higher interpretability than Φ_1_ because it is more representative of the true solution. On the other hand, Φ_1_ and Φ_3_ have similar representativeness, however Φ_3_ is more interpretable due to the higher level of reproducibility.

### 2.4. A heuristic for practical quantification of interpretability in time-locked analysis of MEG data

In practice, it is impossible to evaluate the interpretability, as the true solution of the brain decoding problem Φ^*^ is unknown. In this study, to provide a practical proof of theoretical concepts, we exemplify contrast event-related field (cERF) as a neurophysiological plausible heuristic for the true parameters of the linear brain decoding problem (Θ^*^) in a binary MEG decoding scenario in time domain. Due to the nature of proposed heuristic, its application is limited to the brain responses that are time-locked to the stimulus onset, i.e., the evoked responses.

The MEEG data are a mixture of several simultaneous stimulus-related and stimulus-unrelated brain activities. Assessing the electro/magneto-physiological changes that are time-locked to events of interest is a common approach to the study of MEEG data. In general, unrelated-stimulus brain activities are considered as Gaussian noise with zero mean and variance σ^2^. One popular approach to canceling the noise component is to compute the average of multiple trials. The assumption is that, when the effect of interest is time-locked to the stimulus onset, the independent noise components can be vanished by means of averaging. It is expected that the average will converge to the true value of the signal with a variance of $\frac{\sigma^{2}}{n}$ (where *n* is the number of trials). The result of the averaging process consist of a series of positive and negative peaks occurring at a fixed time relative to the event onset, generally known as ERF in the MEG context. These component peaks are reflecting phasic activity that are indexed with different aspects of cognitive processing (Rugg and Coles, 1995)^1^.

Assume ${\text{X}}^{+}=\left\{x_{i}\in \text{X}|y_{i}=1\right\}\in {\mathbb{R}}^{n^{+}\times p}$ and ${\text{X}}^{-}=\left\{x_{i}\in \text{X}|y_{i}=-1\right\}\in {\mathbb{R}}^{n^{-}\times p}$ be sets of positive and negative samples in a binary MEG decoding scenario. Then, the cERF brain map ${\vec{\Theta }}^{cERF}$ is computed as follows:

(11) $$\begin{array}{l} {\vec{\Theta }}^{cERF}=\frac{\frac{1}{n^{+}}\underset{x_{i}\in X^{+}}{\sum }x_{i}-\frac{1}{n^{-}}\underset{x_{i}\in X^{-}}{\sum }x_{i}}{||\frac{1}{n^{+}}\underset{x_{i}\in X^{+}}{\sum }x_{i}-\frac{1}{n^{-}}\underset{x_{i}\in X^{-}}{\sum }x_{i}|{|}_{2}} \end{array}$$

Generally speaking ${\vec{\Theta }}^{cERF}$ is a contrast ERF map between two experimental conditions. Using the core theory presented in Haufe et al. (2013), the equivalent generative model for the solution of linear brain decoding, i.e., the activation pattern (*A*), is unique and we have:

(12) $$\begin{array}{l} A\propto \Sigma_{\text{X}}\hat{\Theta } \end{array}$$

Assuming $\hat{\Theta }$ to be the solution of least squares in a binary decoding scenario, then the following proposition describes the relation between ${\vec{\Theta }}^{cERF}$ and the activation pattern *A*:

Proposition 2. ${\vec{\Theta }}^{cERF}\propto A$.

See Appendix 6.2 for the proof. Proposition 2.4 shows that, in a binary time-domain MEG decoding scenario, cERF is proportional to the equivalent generative model for the solution of least squares classifier. Furthermore, ${\vec{\Theta }}^{cERF}$ is proportional to the t-statistic that is widely used in the univariate analysis of neuroimaging data. Using ${\vec{\Theta }}^{cERF}$ as a heuristic for ${\vec{\Theta }}^{*}$, the representativeness can be approximated as follows:

(13) $$\begin{array}{l} {\tilde{\beta }}_{\Phi }=\frac{\left|{\vec{\Theta }}^{\mu }.{\vec{\Theta }}^{cERF}\right|}{||{\vec{\Theta }}^{\mu }|{|}_{2}||{\vec{\Theta }}^{cERF}|{|}_{2}} \end{array}$$

Where ${\tilde{\beta }}_{\Phi }$ is an approximation of the actual representativeness β_Φ_. In a similar manner, ${\vec{\Theta }}^{cERF}$ can be used to heuristically approximate the interpretability as follows:

(14) $$\begin{array}{l} {\tilde{\eta }}_{\Phi }=\frac{1}{m}\overset{m}{\underset{j=1}{\sum }}\cos\left(\gamma^{j}\right) \end{array}$$

where γ_1_, …, γ_*m*_ are the angles between ${\vec{\hat{\Theta }}}^{1},\ldots ,{\vec{\hat{\Theta }}}^{m}$ and ${\vec{\Theta }}^{cERF}$. It can be shown that ${\tilde{\eta }}_{\Phi }={\tilde{\beta }}_{\Phi }\times \psi_{\Phi }$.

The proposed heuristic is only applicable to the evoked responses in sensor and source space MEEG data. Despite this limitation, cERF provides an empirical example that shows how the presented theoretical definitions can be applied in a real decoding scenario. The choice of the heuristic has a direct effect on the approximation of interpretability and that an inappropriate selection of the heuristic yields a very poor estimation of interpretability. Therefore, the choice of heuristic should be carefully justified based on accepted and well-defined facts regarding the nature of the collected data.

Since the labels are used in the computation of cERF, a proper validation strategy should be employed to avoid the double dipping issue (Kriegeskorte et al., 2009). One possible approach is to exclude the entire test set from the model selection procedure using a nested nested cross-validation strategy. An alternative approach is employing model averaging techniques to neatly get advantage of the whole dataset (Varoquaux et al., 2017). Since our focus is on the model selection, in the remaining text, we implicitly assume the test data is excluded from the experiments, thus, all the experimental results are reported on the training and validation sets.

### 2.5. Incorporating the interpretability into model selection

The procedure for evaluating the performance of a model so as to choose the best values for hyper-parameters is known as *model selection* (Hastie et al., 2009). This procedure generally involves numerical optimization of the model selection criterion on the training and validation sets (and not the test set). Let *U* be a set of hyper-parameters, then the goal of model selection procedure reduces to finding the best model configuration u^*^ ∈ *U* that maximizes the model selection criterion (e.g., generalization performance) on the training set *S*. The most common model selection criterion is based on an estimator of generalization performance, i.e., the predictive power. In the context of brain decoding, especially when the interpretability of brain maps matters, employing the predictive power as the only decisive criterion in model selection is problematic in terms of interpretability of MBMs (Gramfort et al., 2012; Rasmussen et al., 2012; Conroy et al., 2013; Varoquaux et al., 2017). Valverde-Albacete and Peláez-Moreno (2014) experimentally showed that in a classification task optimizing only classification error rate is insufficient to capture the transfer of crucial information from the input to the output of a classifier. This fact highlights the importance of having some control over the estimated model weights in the model selection. Here, we propose a multi-objective criterion for model selection that takes into account both prediction accuracy and MBM interpretability.

Let ${\tilde{\eta }}_{\Phi }$ and δ_Φ_ be the approximated interpretability and the generalization performance of a linear brain decoding model $\hat{\Phi }$, respectively. We propose the use of the *scalarization* technique (Caramia and Dell´ Olmo, 2008) for combining ${\tilde{\eta }}_{\Phi }$ and δ_Φ_ into one scalar 0 ≤ ζ(Φ) ≤ 1 as follows:

(15) $$\zeta_{\Phi }=\{\begin{array}{cc} \frac{\omega_{1}{\tilde{\eta }}_{\Phi }\;+\;\omega_{2}\delta_{\Phi }}{\omega_{1}\;+\;\omega_{2}} & \delta_{\Phi }\geq \kappa \\ 0 & \delta_{\Phi }<\kappa \end{array}$$

where ω_1_ and ω_2_ are weights that specify the level of importance of the interpretability and the performance, respectively. κ is a threshold on the performance that filters out solutions with poor performance. In classification scenarios, κ can be set by adding a small safe interval to the chance level of classification. The hyper-parameters that are optimized based on ζ_Φ_ are Pareto optimal (Marler and Arora, 2004). We hypothesize that optimizing the hyper-parameters based on ζ_Φ_, rather only δ_Φ_, yields more informative MBMs.

Algorithm 1 summarizes the proposed model selection scheme. The model selection procedure receives the training set *S* and a set of possible configurations for hyper-parameters *U*, and returns the best hyper-parameter configuration u^*^.

**Algorithm 1:** The model selection procedure

1: **procedure** ModelSelection(*S*,*U*)
2: Compute ${\vec{\Theta }}^{cERF}$ on *S*. ⊳ using Equation (11)
3: **for all** *u*_*i*_ ∈ *U* **do** ⊳ For all hyper-parameter configurations.
4: **for** *j* ← 1, *m* **do** ⊳ Data perturbation iterations.
5: Partition *S* into training *S*_*tr*_ and validation *S*_*vl*_
6: subsets via a perturbation method.
7: Compute ${\hat{\Theta }}_{j}$ on *S*_*tr*_ using *u*_*i*_ as the
8: hyper-parameter.
**end**
9: Compute$\delta_{\Phi }^{i}$ of ${\hat{\Theta }}_{j}$s on *S*_*vl*_.
10: Compute ${\tilde{\eta }}_{\Phi }^{i}$ of ${\hat{\Theta }}_{j}$s using ${\vec{\Theta }}^{cERF}$. ⊳ using Equation (14)
11: Compute $\zeta_{\Phi }^{i}$. ⊳ using Equation (15)
**end**
12: u^*^ = argmax_*u*_*i*_ ∈ *U*_(ζ_Φ_).
13: return u^*^.

### 2.6. Experimental materials

#### 2.6.1. Toy dataset

We regenerate the simple 2-dimensional toy data presented in Haufe et al. (2013). Assume that the true underlying generative function Φ^*^ is defined by:

$$Y=\Phi^{*}(X)=\{\begin{array}{cc} 1 & \;if\;x_{1}=1.5\\ -1 & \;if\;x_{1}=-1.5 \end{array}$$

where $X\in \left\{{\left[1.5,0\right]}^{T},{\left[-1.5,0\right]}^{T}\right\}$; and *x*_1_ and *x*_2_ represent the first and the second dimension of the data, respectively. Furthermore, assume the data is contaminated by Gaussian noise with co-variance $\Sigma =\left[\begin{array}{cc} 1.02 & -0.3\\ -0.3 & 0.15 \end{array}\right]$. In fact, the Gaussian noise adds uncertainty to the input space.

#### 2.6.2. Simulated MEG data

**Figure 2:**
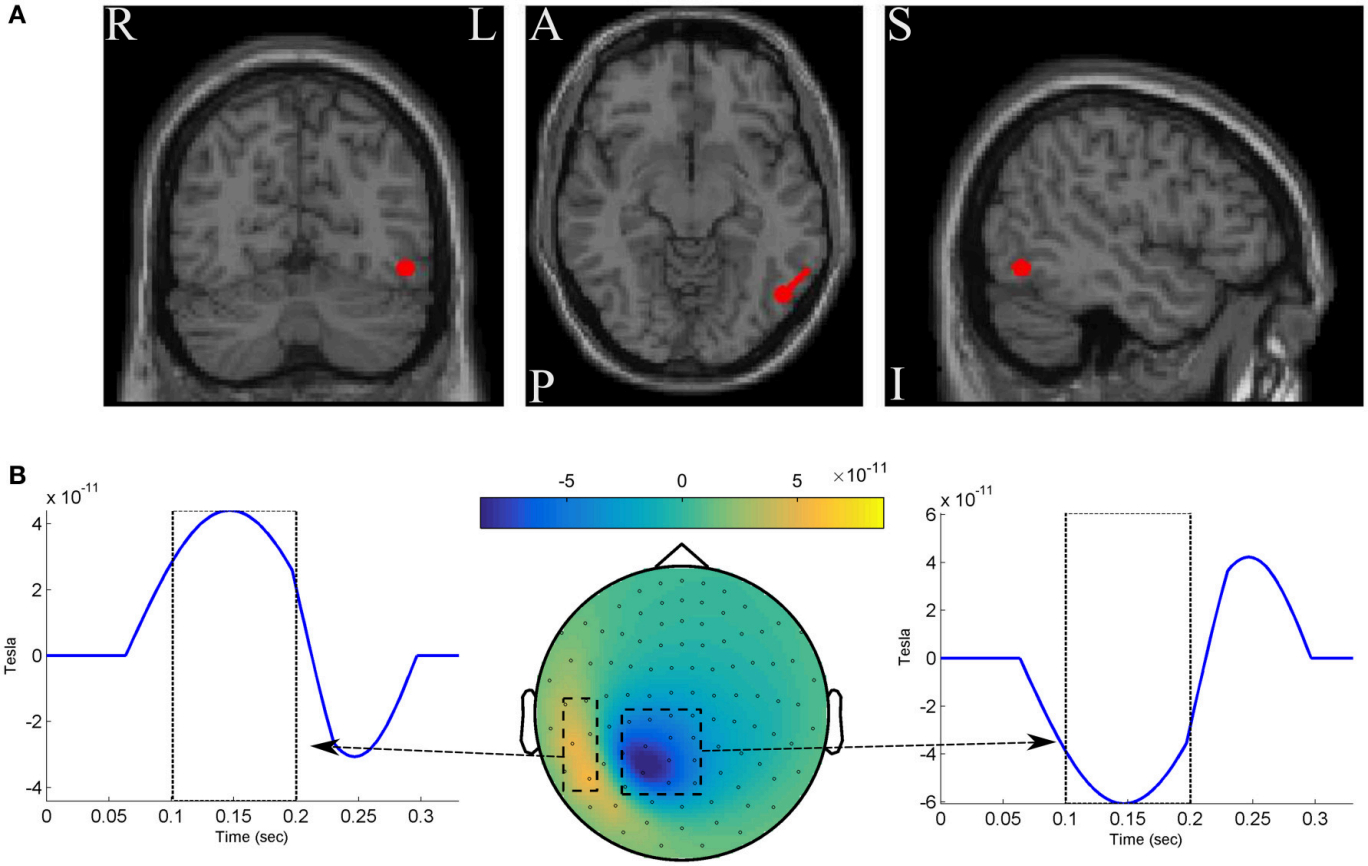
**(A)** The red circles show the dipole position, and the red stick shows the dipole direction. **(B)** The spatio-temporal pattern of the discriminative ground-truth effect.

We simulated two classes of MEG data, each of which composed of 250 epochs with length of 330*ms* at 300*Hz* sampling rate (so that we have 100 time-points). For simplicity, the whole scalp topography are simulated with a single dipole located at −4.7, −3.7, and 5.3*cm* in the RAS (right, anterior, superior) coordinate system. The dipole is oriented toward [1,1,0] direction in the RA plane (see Figure 2A). One hundred two magnetometer sensors of Elekta Neuromag system are simulated using a standard forward model algorithm implemented in the Fieldtrip toolbox (Oostenveld et al., 2010). The epochs of the positive class are constructed by adding three components to the dipole time-course: (1) a time-locked ERF effect with a positive 3*Hz* followed by a negative 5 *Hz* half-cycle sinusoid peaks after 150 ± 10*ms* and 250 ± 10*ms* of the epoch onset, respectively; (2) uncorrelated background brain activity that was simulated by summing 50 sinusoids with random frequency from 1 to 125*Hz*, and random phase varied between 0 and 2π. Following the data simulation procedure in Yeung et al. (2004), the amplitude of any single frequency component of the signal (the ERF effect and the background noise) is set based on the empirical spectral power of human brain activity to mimic the actual magnetic features of scalp surface; and (3) white Gaussian noise scaled with the root mean squared of the signal in each epoch. The epochs of the negative class are constructed without the ERF effect by adding up only the noise components (i.e., the background activity and the white noise). Therefore, the ERF component is considered as the discriminative ground-truth in our experiments (see Figure 2B).

#### 2.6.3. MEG data

We use the MEG dataset presented in Henson et al. (2011)^2^. The dataset was also used for the DecMeg2014 competition^3^. In this dataset, visual stimuli consisting of famous faces, unfamiliar faces, and scrambled faces are presented to 16 subjects and fMRI, EEG, and MEG signals are recorded. Here, we are only interested in MEG recordings. The MEG data were recorded using a VectorView system (Elekta Neuromag, Helsinki, Finland) with a magnetometer and two orthogonal planar gradiometers located at 102 positions in a hemispherical array in a light Elekta-Neuromag magnetically shielded room.

Three major reasons motivated the choice of this dataset: (1) It is publicly available. (2) The spatio-temporal dynamic of the MEG signal for face vs. scramble stimuli has been well studied. The event-related potential analysis of EEG/MEG shows that *N*170 occurs 130 − 200*ms* after stimulus presentation and reflects the neural processing of faces (Bentin et al., 1996; Henson et al., 2011). Therefore, the *N*170 component can be considered the ground truth for our analysis. (3) In the literature, non-parametric mass-univariate analysis such as cluster-based permutation tests is unable to identify narrowly distributed effects in space and time (e.g., an *N*170 component; Groppe et al., 2011a,b). These facts motivate us to employ multivariate approaches that are more sensitive to these effects.

As in Olivetti et al. (2014), we created a balanced face vs. scrambled MEG dataset by randomly drawing from the trials of unscrambled (famous or unfamiliar) faces and scrambled faces in equal number. The samples in the face and scrambled face categories are labeled as 1 and −1, respectively. The raw data is high-pass filtered at 1*Hz*, down-sampled to 250*Hz*, and trimmed from 200*ms* before the stimulus onset to 800*ms* after the stimulus. Thus, each trial has 250 time-points for each of the 306 MEG sensors (102 magnetometers and 204 planar gradiometers)^4^. To create the feature vector of each sample, we pooled all of the temporal data of 306 MEG sensors into one vector (i.e., we have *p* = 250 × 306 = 76500 features for each sample). Before training the classifier, all of the features are standardized to have a mean of 0 and standard-deviation of 1.

### 2.7. Classification and evaluation

In all experiments, Lasso (Tibshirani, 1996b) classifier with ℓ_1_ penalization is used for decoding. Lasso is a very popular classification method in the context of brain decoding, mainly because of its sparsity assumption. The choice of Lasso, as a simple model with only one hyper-parameter, helps us to better illustrate the importance of including the interpretability in the model selection (see the supplementary materials for the results of the elastic-net; Zou and Hastie, 2005 classifier). The solution of decoding is computed by solving the following optimization problem:

(16) $$\begin{array}{l} \hat{\Theta }=\underset{\Theta }{\mathrm{argmin}}L\left(\text{X}\Theta ,\text{Y}\right)+\lambda ||\Theta |{|}_{1} \end{array}$$

where ||.||_1_ represents the ℓ_1_-norm, and λ is the hyper-parameter that specifies the level of regularization. Therefore, the aim of the model selection is to find the best value for λ on the training set *S*. Here, we try to find the best regularization parameter value among λ = {0.001, 0.01, 0.1, 1, 10, 50, 100, 250, 500, 1000}.

We use the out-of-bag (OOB) (Wolpert and Macready, 1999; Breiman, 2001) method for computing δ_Φ_, ψ_Φ_, ${\tilde{\beta }}_{\Phi }$, ${\tilde{\eta }}_{\Phi }$, and ζ_Φ_ for different values of λ. In OOB, given a training set (**X**, **Y**), *m* replications of bootstrap (Efron, 1992) are used to create perturbed training and validation sets (we set *m* = 50)^5^. In all of our experiments, we set ω_1_ = ω_2_ = 1 and κ = 0.6 in the computation of ζ_Φ_. Furthermore, we set δ_Φ_ = 1−*EPE* where EPE indicates the expected prediction error; it is computed using the procedure explained in Appendix 6.4. Employing OOB provides the possibility of computing the bias and variance of the model as contributing factors in EPE.

## 3. Results

### 3.1. Performance-interpretability dilemma: a toy example

In the definition of Φ^*^ on the toy dataset discussed in Section 2.6.1, *x*_1_ is the decisive variable and *x*_2_ has no effect on the classification of samples into target classes. Therefore, excluding the effect of noise and based on the theory of the maximal margin classifier (Vapnik and Kotz, 1982), ${\vec{\Theta }}^{*}\propto {\left[1,0\right]}^{T}$ is the true solution to the decoding problem. By accounting for the effect of noise, solving the decoding problem in (**X**, **Y**) space yields $\vec{\hat{\Theta }}\propto {\left[1/\sqrt{5},2/\sqrt{5}\right]}^{T}$ as the parameters of the linear classifier. Although the estimated parameters on the noisy data provide the best generalization performance for the noisy samples, any attempt to interpret this solution fails, as it yields the wrong conclusion with respect to the ground truth (it says *x*_2_ has twice the influence of *x*_1_ on the results, whereas it has no effect). This simple experiment shows that the most accurate model is not always the most interpretable one, primarily because the contribution of the noise in the decoding process (Haufe et al., 2013). On the other hand, the true solution of the problem ${\vec{\Theta }}^{*}$ does not provide the best generalization performance for the noisy data.

**Figure 3:**
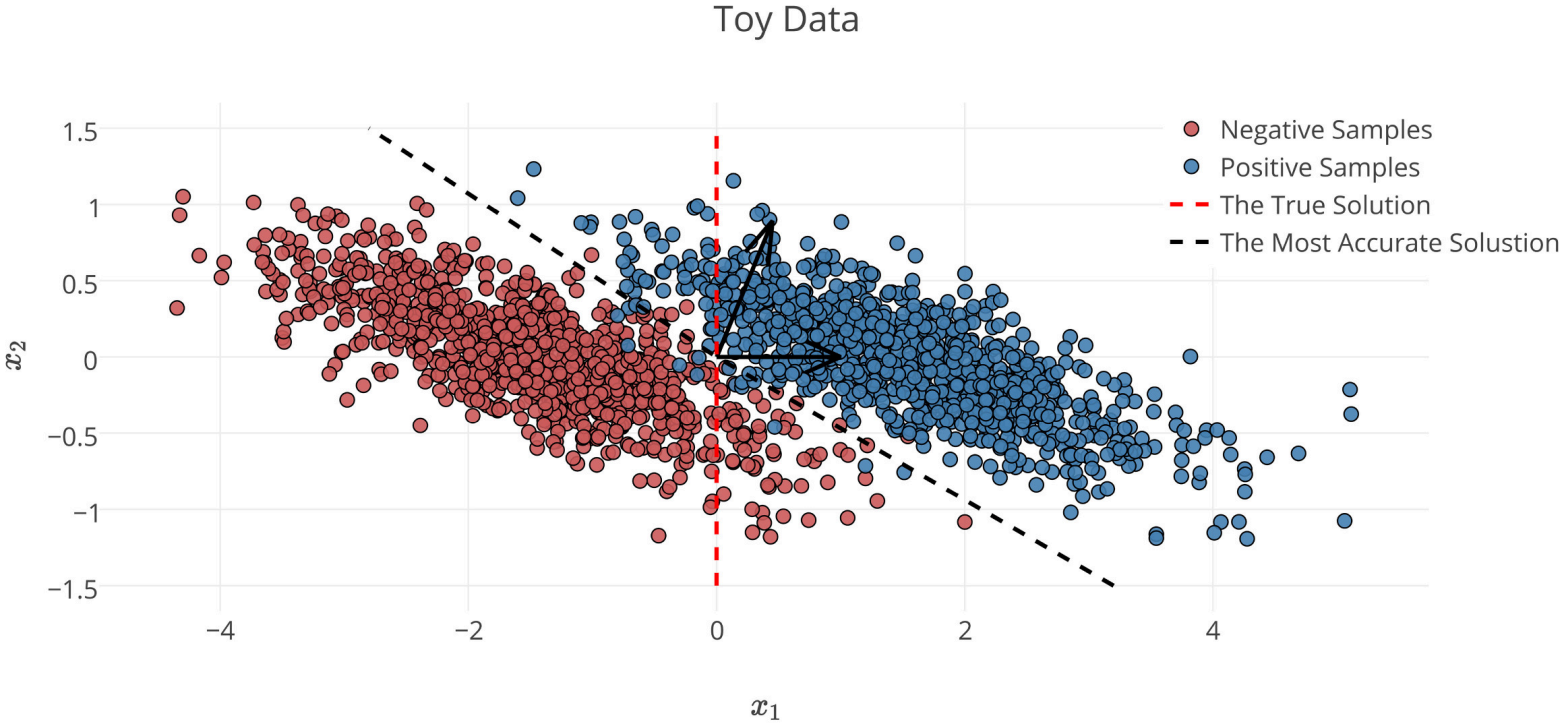
**Noisy samples of toy data**. The red dashed line shows the true separator based on the generative model (Φ^*^). The black dashed line shows the most accurate classification solution. Because of the contribution of noise, any interpretation of the parameters of the most accurate classifier yields a misleading conclusion with respect to the true underlying phenomenon (Haufe et al., 2013).

To illustrate the effect of incorporating the interpretability in the model selection, a Lasso model with different λ values is used for classifying the toy data. In this example, because ${\vec{\Theta }}^{*}$ is known, the exact value of interpretability can be computed using Equation (5). Table 1 compares the resultant performance and interpretability from Lasso. Lasso achieves its highest performance (δ_Φ_ = 0.9884) at λ = 10 with $\vec{\hat{\Theta }}\propto {\left[0.4636,0.8660\right]}^{T}$ (indicated by the black dashed line in Figure 3). Despite having the highest performance, this solution suffers from a lack of interpretability (η_Φ_ = 0.4484). By increasing λ, the interpretability improves so that for λ = 500, 1000 the classifier reaches its highest interpretability by compensating for 0.06 of its performance. Our observation highlights two main points:

1. In the case of noisy data, the interpretability of a decoding model can be possibly incoherent with its performance. Thus, optimizing the parameter of the model based on its performance does not necessarily improve its interpretability. This observation confirms the previous finding by Rasmussen et al. (2012) regarding the trade-off between the spatial reproducibility (as a measure for the interpretability) and the prediction accuracy in brain decoding.
2. If the right criterion is used in the model selection, employing proper regularization technique (sparsity prior, in the case of toy data) leads to more interpretable decoding models.

**Table 1:** **Comparison between δ_Φ_, η_Φ_, and ζ_Φ_ for different λ values on the toy example shows the performance-interpretability dilemma, in which the most accurate classifier is not the most interpretable one**.

**λ**	**0**	**0.001**	**0.01**	**0.1**	**1**	**10**	**50**	**100**	**250**	**500**	**1000**
δ(Φ)	0.9883	0.9883	0.9883	0.9883	0.9883	**0.9884**	0.9880	0.9840	0.9310	0.9292	0.9292
η(Φ)	0.4391	0.4391	0.4391	0.4392	0.4400	0.4484	0.4921	0.5845	0.9968	**1**	**1**
ζ(Φ)	0.7137	0.7137	0.7137	0.7137	0.7142	0.7184	0.7400	0.7842	0.9639	**0.9646**	**0.9646**
$\vec{\hat{\Theta }}\propto$	$\left[\begin{array}{c} 0.4520\\ 0.8920 \end{array}\right]$	$\left[\begin{array}{c} 0.4520\\ 0.8920 \end{array}\right]$	$\left[\begin{array}{c} 0.4520\\ 0.8920 \end{array}\right]$	$\left[\begin{array}{c} 0.4521\\ 0.8919 \end{array}\right]$	$\left[\begin{array}{c} 0.4532\\ 0.8914 \end{array}\right]$	$\left[\begin{array}{c} 0.4636\\ 0.8660 \end{array}\right]$	$\left[\begin{array}{c} 0.4883\\ 0.8727 \end{array}\right]$	$\left[\begin{array}{c} 0.5800\\ 0.8146 \end{array}\right]$	$\left[\begin{array}{c} 0.99\\ 0.02 \end{array}\right]$	$\left[\begin{array}{c} 1\\ 0 \end{array}\right]$	$\left[\begin{array}{c} 1\\ 0 \end{array}\right]$

*The bold indicates the best values of different criteria*.

### 3.2. Decoding on simulated MEG data

**Figure 4:**
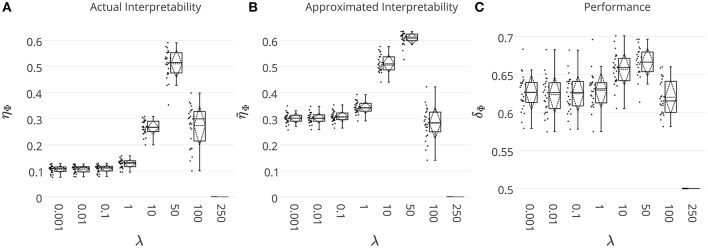
**(A)** The actual η_Φ_, and **(B)** the heuristically approximated interpretability ${\tilde{\eta }}_{\Phi }$ of decoding models across different λ values. There is a significant co-variation (Pearson's correlation p-value = 9 × 10^−4^) between η_Φ_ and ${\tilde{\eta }}_{\Phi }$. **(C)** The generalization performance of decoding models. The box gives the quartiles, while the whiskers give the 5 and 95 percentiles.

With the main aim of comparing the quality of the heuristically approximated interpretability with respect to its actual value, we solve the decoding problem on the simulated MEG data where the ground-truth discriminative effect is known. The ground truth effect ${\vec{\Theta }}^{*}$ is used to compute the actual interpretability of the decoding model. On the other hand, interpretability is approximated by means of ${\vec{\Theta }}^{cERF}$. The whole data simulation and decoding processes are repeated 25 times and the results are summarized in Figure 4. Figures 4A,B show the actual (η_Φ_) and the approximated (${\tilde{\eta }}_{\Phi }$) interpretability for different λ values. Even though ${\tilde{\eta }}_{\Phi }$ consistently overestimates η_Φ_, there is a significant co-variation (Pearson's correlation p-value = 9 × 10^−4^) between two measures as λ increases. Thus, despite overestimation problem of the heuristically approximated interpretability values, they are still reliable measures for quantitative comparison between interpretability level of brain decoding models with different hyper-parameters. For example, both η_Φ_ and ${\tilde{\eta }}_{\Phi }$ suggest the decoding model with λ = 50 as the most interpretable model.

**Figure 5:**
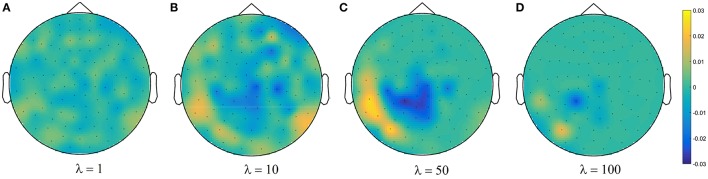
**Topographic maps of weights of brain decoding models for (A)** λ = 1, **(B)** λ = 10, **(C)** λ = 50, and **(D)** λ = 100.

Figure 4C shows brain decoding models at λ = 10 and λ = 50 yield equivalent generalization performances (Wilcoxon rank sum test *p*-value = 0.08), while the MBM resulted from λ = 50 has significantly higher interpretability (Wilcoxon rank sum test *p*-value = 4 × 10^−9^). The advantage of this difference in interpretability levels is visualized in Figure 5 where topographic maps are plotted for the weights of brain decoding models with different λ values by averaging the classifier weights in the time interval of 100–200 ms. The visual comparison shows MBM at λ = 50 is more similar to the ground-truth map (see Figure 2B) than the MBMs computed at other λ values. This superiority is well-reflected in the corresponding approximated interpretability values, that confirms the effectiveness of the interpretability criterion in measuring the level of information in the MBMs.

The results of this experiment confirm again the fact that the generalization performance is not a reliable criterion to measure the level of information learned by a linear classifier. For example consider the decoding model with λ = 1 in which the performance of the model is significantly above the chance level (see Figure 4C) while the corresponding MBM (Figure 5A) is completely misrepresents the ground-truth effect (Figure 2B).

### 3.3. Single-subject decoding on MEG data

To investigate the behavior of the proposed model selection criterion ζ_Φ_, we benchmark it against the commonly used performance criterion δ_Φ_ in a single-subject decoding scenario. Assuming (**X**_*i*_, **Y**_*i*_) for *i* = 1, …, 16 are MEG trial/label pairs for subject *i*, we separately train a Lasso model for each subject to estimate the parameter of the linear function ${\hat{\Phi }}_{i}$, where ${\text{Y}}_{i}={\text{X}}_{i}{\hat{\Theta }}_{i}$. We represent the optimized solution based on δ_Φ_ and ζ_Φ_ by ${\hat{\Phi }}_{i}^{\delta }$ and ${\hat{\Phi }}_{i}^{\zeta }$, respectively. We also denote the MBM associated with ${\hat{\Phi }}_{i}^{\delta }$ and ${\hat{\Phi }}_{i}^{\zeta }$ by ${\vec{\hat{\Theta }}}_{i}^{\delta }$ and ${\vec{\hat{\Theta }}}_{i}^{\zeta }$, respectively. Therefore, for each subject, we compare the resulting decoders and MBMs computed based on these two model selection criteria.

**Figure 6:**
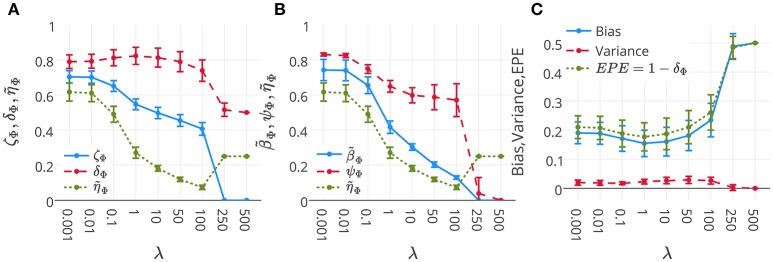
**(A)** Mean and standard-deviation of the performance (δ_Φ_), interpretability (η_Φ_), and ζ_Φ_ of Lasso over 16 subjects. **(B)** Mean and standard-deviation of the reproducibility (ψ_Φ_), representativeness (β_Φ_), and interpretability (η_Φ_) of Lasso over 16 subjects. The interpretability declines because of the decrease in both reproducibility and representativeness (see Proposition 1). **(C)** Mean and standard-deviation of the bias, variance, and EPE of Lasso over 16 subjects. While the change in bias is correlated with that of EPE (Pearson's correlation coefficient = 0.9993), there is anti-correlation between the trend of variance and EPE (Pearson's correlation coefficient = −0.8884).

Figure 6A represents the mean and standard-deviation of the performance and interpretability of Lasso across 16 subjects for different λ values. The performance and interpretability curves further illustrate the performance-interpretability dilemma of Lasso classifier in the single-subject decoding scenario, in which increasing the performance delivers less interpretability. The average performance across subjects is improved when λ approaches 1, but on the other side, the reproducibility and the representativeness of models declines significantly (see Figure 6B; Wilcoxon rank sum test *p*-value = 9 × 10^−4^ and 8 × 10^−7^, respectively). In fact, in this dataset a higher amount of sparsity increases the gap between the generalization performance and interpretability.

One possible reason behind the performance-interpretability dilemma in this experiment is illustrated in Figure 6C. The figure shows the mean and standard deviation of bias, variance, and EPE of Lasso across 16 subjects. The plot shows while the change in bias is correlated with that of EPE (Pearson's correlation coefficient = 0.9993), there is anti-correlation between the trends of variance and EPE (Pearson's correlation coefficient = −0.8884). Furthermore, it proposes that the effect of variance is overwhelmed by bias in the computation of EPE, where the best performance (minimum EPE) at λ = 1 has the lowest bias, its variance is higher than for λ = 0.001, 0.01, 0.1. While this tiny increase in the variance has negligible effect on the EPE of the model, Figure 6B shows its significant (Wilcoxon rank sum test *p*-value = 8 × 10^−7^) negative effect on the reproducibility of maps from λ = 0.1 to λ = 1.

Table 2 summarizes the performance, reproducibility, representativeness, and interpretability of ${\hat{\Phi }}_{i}^{\delta }$ and ${\hat{\Phi }}_{i}^{\zeta }$ for 16 subjects. The average result over 16 subjects shows that employing ζ_Φ_ instead of δ_Φ_ in model selection provides higher reproducibility, representativeness, and (as a result) interpretability compensating for 0.04 of performance. The last column of table (δ_*cERF*_) summarizes the performance of decoding models over 16 subjects when ${\vec{\Theta }}^{cERF}$ is used as classifier weights. The comparison illustrates a significant difference (Wilcoxon rank sum test *p*-value = 1.5 × 10^−6^) between δ_*cERF*_ and δ(Φ)s. These facts demonstrate that ${\vec{\hat{\Theta }}}^{\zeta }$ is a good compromise between ${\vec{\hat{\Theta }}}_{\delta }$ and ${\vec{\Theta }}^{cERF}$ in terms of classification performance and model interpretability.

**Table 2:** **The performance, reproducibility, representativeness, and interpretability of ${\hat{\Phi }}_{i}^{\delta }$ and ${\hat{\Phi }}_{i}^{\zeta }$ over 16 subjects**.

**Subs**	**Criterion: δ(Φ)**					**Criterion: ζ(Φ)**					**δ_*cERF*_**
	**δ(Φ)**	**ζ(Φ)**	**$\tilde{\eta }\left(\Phi \right)$**	**$\tilde{\beta }\left(\Phi \right)$**	**ψ(Φ)**	**δ(Φ)**	**ζ(Φ)**	**$\tilde{\eta }\left(\Phi \right)$**	**$\tilde{\beta }\left(\Phi \right)$**	**ψ(Φ)**	
1	0.81	0.53	0.26	0.42	0.62	0.78	0.70	0.63	0.76	0.83	0.56
2	0.80	0.70	0.60	0.72	0.83	0.80	0.70	0.60	0.72	0.83	0.54
3	0.81	0.63	0.45	0.64	0.71	0.78	0.71	0.64	0.78	0.83	0.57
4	0.84	0.52	0.20	0.31	0.66	0.76	0.70	0.64	0.77	0.83	0.55
5	0.80	0.54	0.29	0.44	0.65	0.78	0.69	0.61	0.73	0.83	0.54
6	0.79	0.52	0.24	0.39	0.63	0.74	0.67	0.61	0.74	0.82	0.57
7	0.84	0.55	0.27	0.40	0.66	0.81	0.70	0.59	0.71	0.84	0.56
8	0.87	0.55	0.24	0.35	0.68	0.85	0.68	0.52	0.61	0.84	0.56
9	0.80	0.55	0.31	0.46	0.67	0.77	0.67	0.57	0.69	0.82	0.57
10	0.79	0.53	0.26	0.41	0.64	0.77	0.68	0.58	0.70	0.83	0.59
11	0.74	0.65	0.56	0.68	0.82	0.74	0.65	0.56	0.68	0.82	0.53
12	0.80	0.55	0.29	0.46	0.64	0.79	0.70	0.61	0.74	0.83	0.58
13	0.83	0.50	0.18	0.29	0.61	0.77	0.70	0.63	0.76	0.82	0.59
14	0.90	0.58	0.27	0.39	0.68	0.81	0.78	0.74	0.89	0.84	0.62
15	0.92	0.63	0.34	0.48	0.71	0.89	0.78	0.66	0.77	0.86	0.63
16	0.87	0.55	0.23	0.37	0.62	0.81	0.74	0.67	0.81	0.83	0.65
Mean	**0.83**±**0.05**	0.57 ± 0.05	0.31 ± 0.12	0.45 ± 0.13	0.68 ± 0.07	0.79 ± 0.04	**0.70** ± **0.04**	**0.62** ± **0.05**	**0.74** ± **0.06**	**0.83** ± **0.01**	0.58 ± 0.03

*The bold indicates the best mean values over different criteria*.

**Figure 7:**
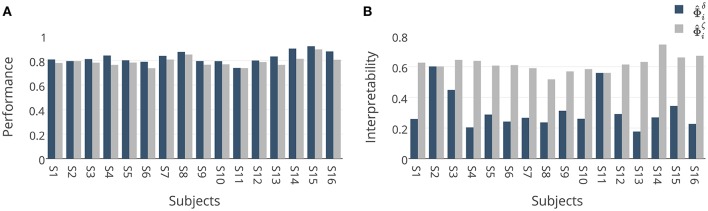
**(A)** Comparison between generalization performances of ${\hat{\Phi }}_{i}^{\delta }$ and ${\hat{\Phi }}_{i}^{\zeta }$. Adopting ζ_Φ_ instead of δ_Φ_ in model selection yields (on average) 0.04 less accurate classifiers over 16 subjects. **(B)** Comparison between interpretabilities of ${\hat{\Phi }}_{i}^{\delta }$ and ${\hat{\Phi }}_{i}^{\zeta }$. Adopting ζ_Φ_ instead of δ_Φ_ in model selection yields on average 0.31 more interpretable classifiers over 16 subjects.

These results are further analyzed in Figure 7 where ${\hat{\Phi }}_{i}^{\delta }$ and ${\hat{\Phi }}_{i}^{\zeta }$ are compared subject-wise in terms of their performance and interpretability. The comparison shows that adopting ζ_Φ_ instead of δ_Φ_ as the criterion for model selection yields higher interpretable models by compensating a negligible degree of performance in 14 out of 16 subjects. Figure 7A shows that employing δ_Φ_ provides on average slightly higher accurate models (Wilcoxon rank sum test *p*-value = 0.012) across subjects (0.83 ± 0.05) than using ζ_Φ_ (0.79 ± 0.04). On the other side, Figure 7B shows that employing ζ_Φ_ and compensating by 0.04 in the performance provides (on average) substantially higher (Wilcoxon rank sum test p-value= 5.6 × 10^−6^) interpretability across subjects (0.62 ± 0.05) compared to δ_Φ_ (0.31 ± 0.12). For example, in the case of subject 1 (see Table 2), using δ_Φ_ in model selection to select the best λ value for the Lasso yields a model with δ_Φ_ = 0.81 and ${\tilde{\eta }}_{\Phi }=0.26$. In contrast, using ζ_Φ_ delivers a model with δ_Φ_ = 0.78 and ${\tilde{\eta }}_{\Phi }=0.63$. This inverse relationship between performance and interpretability is direct consequence of over-fitting of model to the noise structure in a small-sample size brain decoding problem (see Section 3.1).

**Figure 8:**
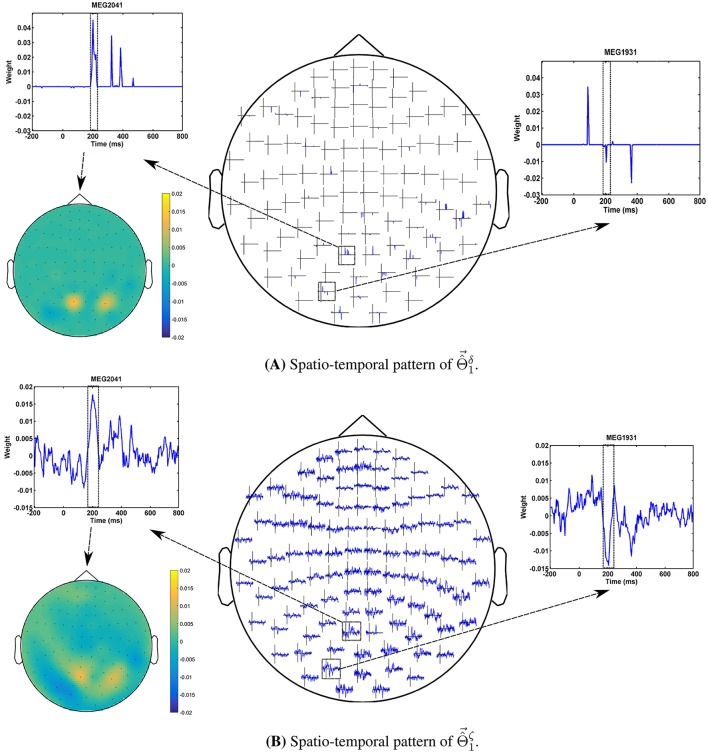
**Comparison between spatio-temporal multivariate maps of (A)** the most accurate, and **(B)** the most interpretable classifiers for Subject 1. ${\vec{\hat{\Theta }}}_{1}^{\zeta }$ provides a better spatio-temporal representation of the N170 effect than ${\vec{\hat{\Theta }}}_{1}^{\delta }$.

The advantage of the exchange between the performance and the interpretability can be seen in the quality of MBMs. Figures 8A,B show ${\vec{\hat{\Theta }}}_{1}^{\delta }$ and ${\vec{\hat{\Theta }}}_{1}^{\zeta }$ of subject 1, i.e., the spatio-temporal multivariate maps of the Lasso models with maximum values of δ_Φ_ and ζ_Φ_, respectively. The maps are plotted for 102 magnetometer sensors. In each case, the time course of weights of classifiers associated with the MEG2041 and MEG1931 sensors are plotted. Furthermore, the topographic maps represent the spatial patterns of weights averaged between 184*ms* and 236*ms* after stimulus onset. While ${\vec{\hat{\Theta }}}_{1}^{\delta }$ is sparse in time and space, it fails to accurately represent the spatio-temporal dynamic of the N170 component. Furthermore, the multicollinearity problem arising from the correlation between the time course of the MEG2041 and MEG1931 sensors causes extra attenuation of the N170 effect in the MEG1931 sensor. Therefore, the model is unable to capture the spatial pattern of the dipole in the posterior area. In contrast, ${\vec{\hat{\Theta }}}_{1}^{\zeta }$ represents the dynamic of the N170 component in time. In addition, it also shows the spatial pattern of two dipoles in the posterior and temporal areas. In summary, ${\vec{\hat{\Theta }}}_{1}^{\zeta }$ suggests a more representative pattern of the underlying neurophysiological effect than ${\vec{\hat{\Theta }}}_{1}^{\delta }$.

**Figure 9:**
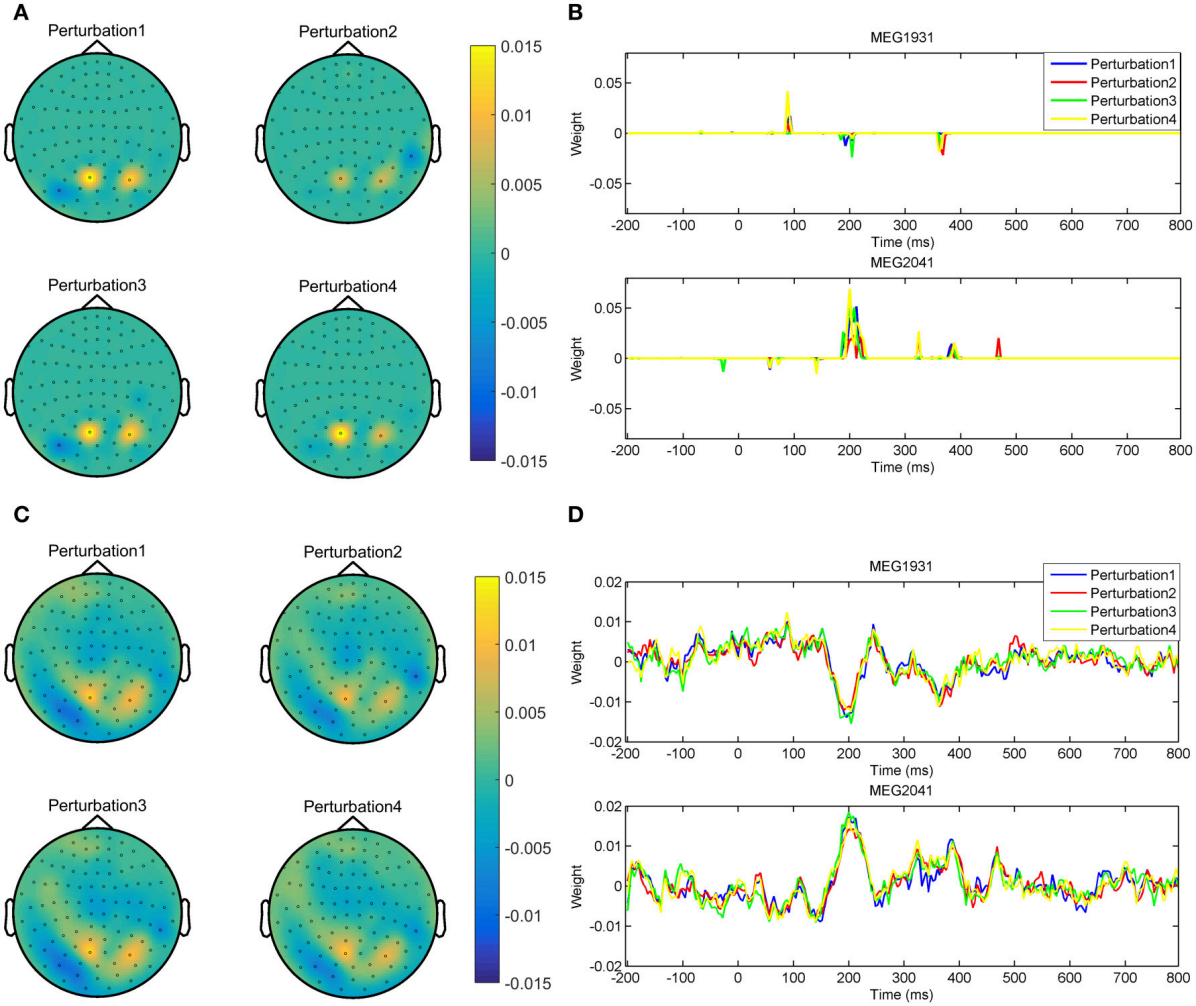
**Comparison of the reproducibility of Lasso when δ_Φ_ and ζ_Φ_ are used in the model selection procedure**. **(A,B)** show the spatio-temporal patterns represented by ${\vec{\hat{\Theta }}}_{1}^{\delta }$ across the 4 perturbed training sets. **(C,D)** show the spatio-temporal patterns represented by ${\vec{\hat{\Theta }}}_{1}^{\zeta }$ across the 4 perturbed training sets. Employing ζ_Φ_ instead of δ_Φ_ in the model selection yields on average 0.15 more reproduciblilty of MBMs.

In addition, optimizing the hyper-parameters of brain decoding based on ζ_Φ_ offers more reproducible brain decoders. According to Table 2, using ζ_Φ_ instead of δ_Φ_ provides (on average) 0.15 more reproducibility over 16 subjects. To illustrate the advantage of higher reproducibility on the interpretability of maps, Figure 9 visualizes ${\vec{\hat{\Theta }}}_{1}^{\delta }$ and ${\vec{\hat{\Theta }}}_{1}^{\zeta }$ over 4 perturbed training sets. The spatial maps (Figures 9A,C) are plotted for the magnetometer sensors averaged in the time interval between 184*ms* and 236*ms* after stimulus onset. The temporal maps (Figures 9B,D) are showing the multivariate temporal maps of MEG1931 and MEG2041 sensors. While ${\vec{\hat{\Theta }}}_{1}^{\delta }$ is unstable in time and space across the 4 perturbed training sets, ${\vec{\hat{\Theta }}}_{1}^{\zeta }$ provides more reproducible maps.

### 3.4. Mass-univariate hypothesis testing on MEG data

It is shown by Groppe et al. (2011a,b) that non-parametric mass-univariate analysis is unable to detect narrowly distributed effects in space and time (e.g., an *N*170 component). To illustrate the advantage of the proposed decoding framework for spotting these effects, we performed a non-parametric cluster-based permutation test (Maris and Oostenveld, 2007) on our MEG dataset using Fieldtrip toolbox (Oostenveld et al., 2010). In a single subject analysis scenario, we considered the trials of MEG recordings as the unit of observation in a between-trials experiment. Independent-samples t-statistics are used as the statistics for evaluating the effect at the sample level and to construct spatio-temporal clusters. The maximum of the cluster-level summed *t*-value is used for the cluster level statistics; the significance probability is computed using a Monte Carlo method. The minimum number of neighboring channels for computing the clusters is set to 2. Considering 0.025 as the two-sided threshold for testing the significance level and repeating the procedure separately for magnetometers and combined-gradiometers, no significant result is found for any of the 16 subjects. This result motivates the search for more sensitive (and, at the same time, more interpretable) alternatives for univariate hypothesis testing.

## 4. Discussions

### 4.1. Defining interpretability: theoretical advantages

An overview of the brain decoding literature shows frequent co-occurrence of the terms interpretation, interpretable, and interpretability with the terms model, classification, parameter, decoding, method, feature, and pattern (see the quick meta-analysis on the literature in the supplementary material); however, a formal formulation of the interpretability is never presented. In this study, our primary interest is to present a simple and theoretical definition of the interpretability of linear brain decoding models and their corresponding MBMs. Furthermore, we show the way in which interpretability is related to the reproducibility and neurophysiological representativeness of MBMs. Our definition and quantification of interpretability remains theoretical, as we assume that the true solution of the brain decoding problem is available. Despite this limitation, we argue that the presented definition provides a concrete framework of a previously abstract concept and that it establishes a theoretical background to explain an ambiguous phenomenon in the brain decoding context. We support our argument using an example in the time-domain MEG decoding in which we show how the presented definition can be exploited to heuristically approximate the interpretability. Our experimental results on MEG data shows accounting for the approximated measure of interpretability has a positive effect on the human interpretation of brain decoding models. This example shows how partial prior knowledge regarding the timing and location of neural activity can be used to find more plausible multivariate patterns in data. Furthermore, the proposed decomposition of the interpretability of MBMs into their reproducibility and representativeness explains the relationship between the influential cooperative factors in the interpretability of brain decoding models and highlights the possibility of indirect and partial evaluation of interpretability by measuring these effective factors.

### 4.2. Application in model evaluation

Discriminative models in the framework of brain decoding provide higher sensitivity and specificity than univariate analysis in hypothesis testing of neuroimaging data. Although multivariate hypothesis testing is performed based solely on the generalization performance of classifiers, the emergent need for extracting reliable complementary information regarding the underlying neuronal activity motivated a considerable amount of research on improving and assessing the interpretability of classifiers and their associated MBMs. Despite ubiquitous use, the generalization performance of classifiers is not a reliable criterion for assessing the interpretability of brain decoding models (Rasmussen et al., 2012; Varoquaux et al., 2017). Therefore, considering extra criteria might be required. However, because of the lack of a formal definition for interpretability, different characteristics of linear classifiers are considered as the decisive criterion in assessing their interpretability. Reproducibility (Rasmussen et al., 2012; Conroy et al., 2013), stability selection (Varoquaux et al., 2012; Wang et al., 2015), sparsity (Dash et al., 2015; Shervashidze and Bach, 2015), and neurophysiological plausibility (Afshin-Pour et al., 2011) are examples of related criteria.

Our definition of interpretability helped us to fill this gap by introducing a new multi-objective model selection criterion as a weighted compromise between interpretability and generalization performance of linear models. Our experimental results on single-subject decoding showed that adopting the new criterion for optimizing the hyper-parameters of brain decoding models is an important step toward reliable visualization of learned models from neuroimaging data. It is not the first time in the neuroimaging context that a new metric is proposed in combination with generalization performance for the model selection. Several recent studies proposed the combination of the reproducibility of the maps (Rasmussen et al., 2012; Conroy et al., 2013; Strother et al., 2014) or the stability of the classifiers (Yu, 2013; Lim and Yu, 2016; Varoquaux et al., 2017) with the performance of discriminative models to enhance the interpretability of decoding models. Our definition of interpretability supports the claim that the reproducibility is not the only effective factor in interpretability. Therefore, our contribution can be considered a complementary effort with respect to the state of the art of improving the interpretability of brain decoding at the model selection level.

Furthermore, this work presents an effective approach for evaluating the quality of different regularization strategies for improving the interpretability of MBMs. As briefly reviewed in Section 1, there is a trend of research within the brain decoding context in which the prior knowledge is injected into the decoding process via the penalization term in order to improve the interpretability of decoding models. Thus far, in the literature, there is no *ad-hoc* method to directly compare the interpretability of MBMs resulting from different penalization techniques. Our findings provide a further step toward direct evaluation of interpretability of the currently proposed penalization strategies. Such an evaluation can highlight the advantages and disadvantages of applying different strategies on different data types and facilitates the choice of appropriate methods for a certain application.

### 4.3. Regularization and interpretability

Haufe et al. (2013) demonstrated that the weight in linear discriminative models are unable to accurately assess the relationship between independent variables, primarily because of the contribution of noise in the decoding process. The authors concluded that the interpretability of brain decoding cannot be improved using regularization. The problem is primarily caused by the decoding process *per se*, where it minimizes the classification error only considering the uncertainty in the output space (Zhang, 2005; Aggarwal and Yu, 2009; Tzelepis et al., 2015) and not the uncertainty in the input space (or noise). Our experimental results on the toy data (see Section 3.1) shows that if the right criterion is used for selecting the best values for hyper-parameters, appropriate choice of the regularization strategy can still play a significant role in improving the interpretability of results. For example, in the case of toy data, the true generative function behind the sampled data is sparse (see Section 2.6.1), but because of the noise in the data, the sparse model is not the most accurate one. On the other hand, a more comprehensive criterion (in this case, ζ_Φ_) that considers also the interpretability of model parameters facilitates the selection of correct prior assumptions about the distribution of the data via regularization. This observation encourages the modification of the conclusion in Haufe et al. (2013) as follows: if the performance of the model is the only criterion in the model selection, then the interpretability cannot necessarily be improved by means of regularization. This modification offers a practical shift in methodology, where we propose to replace the post-processing of weights proposed in Haufe et al. (2013) with refinement of hyper-parameter selection based on the newly developed model selection criterion.

### 4.4. The performance-interpretability dilemma

The performance-interpretability dilemma refers to the trade-off between the generalization performance and the interpretability of a decoding model. In some applications of brain decoding, such as BCI, a more accurate model (even with no interpretability) is desired. On the other hand, when the brain decoding is employed for hypothesis testing purpose, an astute balance between two factors is more favorable. The presented metric for model selection (ζ_Φ_) provides the possibility to maintain this balance. An important question at this point is on the nature of the performance-interpretability dilemma, whether it is model-driven or data-driven? In other words, whether some decoding models (e.g., sparse models) suffer from this deficit, or it is independent from the decoding model and depends on the distribution of data rather assumptions of the decoding model.

Our experiments shed light on the fact that the performance-interpretability dilemma is driven by the *uncertainty* (Aggarwal and Yu, 2009) in data. The uncertainty in data refers to the difference between the true solution of decoding Φ^*^ and the solution of decoding in sampled data space Φ_*S*_, and is generally consequence of noise in the input or/and output spaces. This gap between Φ^*^ and Φ_*S*_ is also known as irreducible error (see Equation 2) in the learning theory, and it cannot fundamentally be reduced by minimizing the error. Therefore, any attempt toward improving the classification performance in the sampled data space might increase the irreducible error. As an example, our experiment on the toy data (see Section 3.1) shows the effect of noise in input space on the performance-interpretability dilemma. Improving the performance of the model (i.e., fitting to Φ_*S*_) diverges the estimated solution of decoding $\hat{\Phi }$ from its true solution Φ^*^, thus reduces the interpretability of the decoding model. Furthermore, our experiments demonstrate that incorporating the interpretability of decoding models in model selection facilitates finding the best match between the decoding model and the distribution of data. For example in classification of toy data, the new model selection metric ζ_Φ_ selects the more sparse model with a better match to the true distribution of data, despite worse generalization performance.

### 4.5. Advantage over mass-univariate analysis

Mass-univariate hypothesis testing methods are among the most popular tools for forward inference on neuroimaging data in cognitive neuroscience field. Mass-univariate analyses consist of univariate statistical tests on single independent variables followed by multiple comparison correction. Generally, multiple comparison correction reduces the sensitivity of mass-univariate approaches because of the large number of univariate tests involved. Cluster-based permutation testing (Maris and Oostenveld, 2007) provides a more sensitive univariate analysis framework by making the cluster assumption in the multiple comparison correction. Unfortunately, this method is not able to detect narrow spatio-temporal effects in the data (Groppe et al., 2011a). As a remedy, brain decoding provides a very sensitive tool for hypothesis testing; it has the ability to detect multivariate patterns, but suffers from a low level of interpretability. Our study proposes a possible solution for the interpretability problem of classifiers, and therefore, it facilitates the application of brain decoding in the analysis of neuroimaging data. Our experimental results for the MEG data demonstrate that, although the non-parametric cluster-based permutation test is unable to detect the N170 effect in MEG data, employing ζ_Φ_ instead of δ_Φ_ in model selection not only detects the stimuli-relevant information in the data, but also assures both reproducible and representative spatio-temporal mapping of the timing and the location of underlying neurophysiological effect.

### 4.6. Limitations and future directions

Despite theoretical and practical advantages, the proposed definition and quantification of interpretability suffer from some limitations. All of the presented concepts are defined for linear models, with the main assumption that $\Phi^{*}\in H$ (where H is a class of linear functions). This fact highlights the importance of linearizing the experimental protocol in the data collection phase (Naselaris et al., 2011). Extending the definition of interpretability to non-linear models demands future research into the visualization of non-linear models in the form of brain maps. Currently, our findings cannot be directly applied to non-linear models. Furthermore, the proposed heuristic for the time-domain MEG data applies only to binary classification. One possible solution in multiclass classification is to separate the decoding problem into several binary sub-problems. In addition the quality of the proposed heuristic is limited for the small sample size datasets. Of course the proposed heuristic is just an example of possible options for assessing the neurophysiological plausibility of MBMs in time-locked analysis of MEG data, thus, improving the quality of heuristic would be of interest in future researches. Finding physiologically relevant heuristics for other acquisition modalities such as fMRI, or frequency domain MEEG data, can be also considered as possible directions in future work.

## 5. Conclusions

We presented a novel theoretical definition for the interpretability of linear brain decoding and associated multivariate brain maps. We demonstrated how the interpretability relates to the representativeness and reproducibility of brain decoding. Although it is theoretical, the presented definition provides a first step toward practical solution for filling the knowledge extraction gap in linear brain decoding. As an example of this major breakthrough, and to provide a proof of concept, a heuristic approach based on the contrast event-related field is proposed for practical evaluation of the interpretability in multivariate recovery of evoked MEG responses. We experimentally showed that adding the interpretability of brain decoding models as a criterion in the model selection procedure yields significantly higher interpretable models by sacrificing a negligible amount of performance. Our methodological and experimental achievements can be considered a complementary theoretical and practical effort that contributes to researches on enhancing the interpretability of multivariate pattern analysis.

## Author contributions

SK contributed in developing the theoretical and experimental contents of this study. SV and AP were involved in developing the theoretical machine learning aspects. NW was advising on the MEG experimental aspects.

### Conflict of interest statement

The authors declare that the research was conducted in the absence of any commercial or financial relationships that could be construed as a potential conflict of interest.

## Figures and Tables

**Figure A1:**
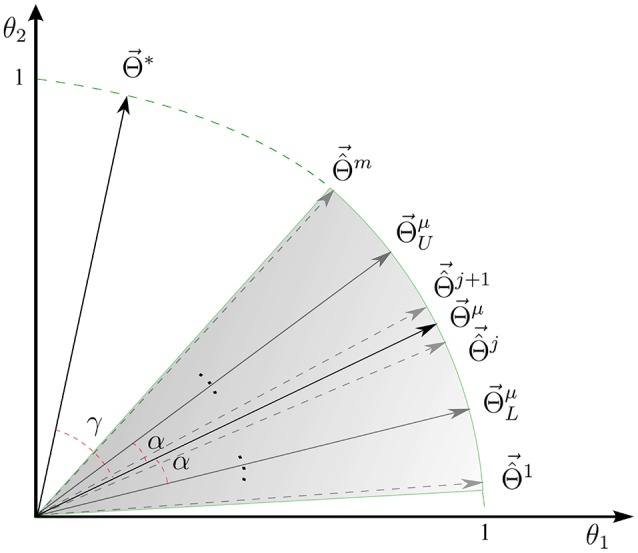
**Relation between representativeness, reproducibility, and interpretability in two dimensions**.

**Figure A2:**
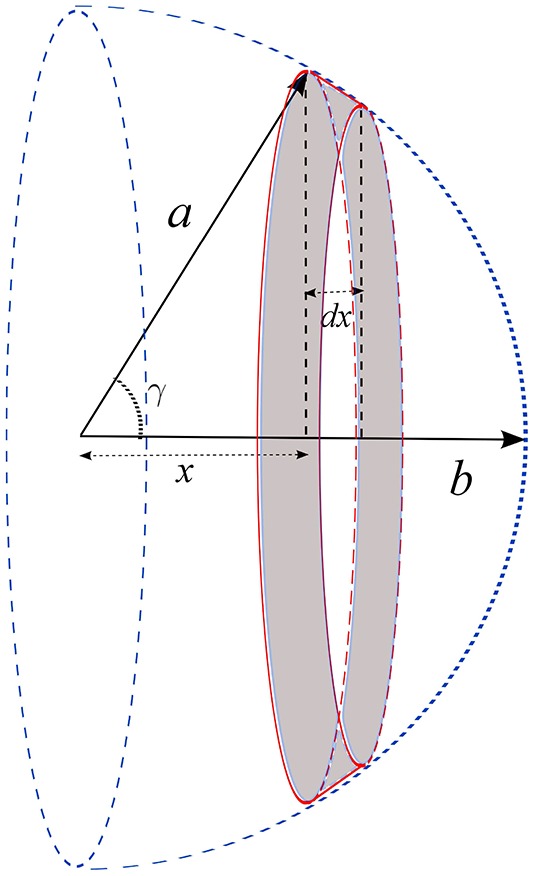
**Two-dimensional geometrical illustration for computing the PDF of cosine similarity**.

## Footnotes

^1^The application of the presented heuristic to MEG data can be extended to EEG because of the inherent similarity of the measured neural correlates in these two devices. In the EEG context, the ERF can be replaced by the event-related potential (ERP).

^2^The full dataset is publicly available at ftp://ftp.mrc-cbu.cam.ac.uk/personal/rik.henson/wakemandg_hensonrn/.

^3^The competition data are available at http://www.kaggle.com/c/decoding-the-human-brain.

^4^The preprocessing scripts in python and MATLAB are available at: https://github.com/FBK-NILab/DecMeg2014/.

^5^The MATLAB code used for experiments is available at https://github.com/smkia/interpretability/.

## A. Appendices

### A.1. Proof of proposition 1

Throughout this proof, we assume that all of the parameter vectors are normalized in the unit hypersphere (see Figure A1 as an illustrative example in two dimensions). Let $T=\left\{{\vec{\hat{\Theta }}}^{1},\ldots ,{\vec{\hat{\Theta }}}^{m}\right\}$ be a set *m* MBMs, for *m* perturbed training sets where ${\vec{\hat{\Theta }}}^{i}\in {\mathbb{R}}^{p}$. Now, consider any arbitrary *p* − 1 dimensional hyperplane A that contains ${\vec{\Theta }}^{\mu }$. Clearly, A divides the *p*-dimensional parameter space into two subspaces. Let ▽ and ▼ be binary operators where ${\vec{\Theta }}^{i}▽{\vec{\Theta }}^{k}$ indicates that ${\vec{\Theta }}^{i}$ and ${\vec{\Theta }}^{k}$ are in the same subspace, and ${\vec{\Theta }}^{i}▼{\vec{\Theta }}^{k}$ indicates that they are in different subspaces. Now, we define $T_{U}=\left\{{\vec{\Theta }}^{i}|{\vec{\Theta }}^{i}▽{\vec{\Theta }}^{*}\right\}$ and $T_{L}=\left\{{\vec{\Theta }}^{i}|{\vec{\Theta }}^{i}▼{\vec{\Theta }}^{*}\right\}$. Let the cardinality of *T*_*L*_ denoted by *n*(*T*_*L*_) be *j* (*n*(*T*_*L*_) = *j*). Thus, *n*(*T*_*U*_) = *m* − *j*. Now, assume that $∡\left({\vec{\hat{\Theta }}}^{i},A\right)=\alpha_{1},\ldots ,\alpha_{j}$ are the angles between ${\vec{\hat{\Theta }}}^{i}\in T_{L}$ and A, and (similarly) α_*j*+1_, …, α_*m*_ for ${\vec{\hat{\Theta }}}^{i}\in T_{U}$ and A. Based on Equation (6), let ${\vec{\Theta }}_{L}^{\mu }$ and ${\vec{\Theta }}_{U}^{\mu }$ be the main maps of *T*_*L*_ and *T*_*U*_, respectively. Therefore, we obtain ${\vec{\Theta }}^{\mu }=\frac{{\vec{\Theta }}_{L}^{\mu }+{\vec{\Theta }}_{U}^{\mu }}{\left||{\vec{\Theta }}_{L}^{\mu }+{\vec{\Theta }}_{U}^{\mu }|\right|}$ and $∡\left({\vec{\Theta }}_{L}^{\mu },A\right)=∡\left({\vec{\Theta }}_{U}^{\mu },A\right)=\alpha$. Furthermore, assume $∡\left({\vec{\Theta }}^{*},A\right)=\gamma$. As a result, ψ_Φ_ = cos(α) and β_Φ_ = cos(γ). According to Equation (4) and using a cosine similarity definition, we have:

(A1) $$\begin{array}{l} \eta_{\Phi }=\frac{1}{m}\sum_{j=1}^{m}\left|{\vec{\Theta }}^{*}.{\vec{\hat{\Theta }}}^{j}\right|\\ \;=\frac{\begin{array}{l} \cos(\gamma +\alpha_{1})+\ldots +\cos(\gamma +\alpha_{j})+\cos(\gamma -\alpha_{j+1})+\ldots \\ \;+\cos(\gamma -\alpha_{m}) \end{array}}{m}\\ \;=\frac{\cos(\gamma +\alpha )+\cos(\gamma -\alpha )}{2}\\ \;=\frac{\begin{array}{l} \cos(\gamma )\cos(\alpha )-\sin(\gamma )\sin(\alpha )+\cos(\gamma )\cos(\alpha )\\ \;+\sin(\gamma )\sin(\alpha ) \end{array}}{2}\\ \;=\cos(\gamma )\cos(\alpha )=\beta_{\Phi }\times \psi_{\Phi }. \end{array}$$

A similar procedure can be used to prove ${\tilde{\eta }}_{\Phi }={\tilde{\beta }}_{\Phi }\times \psi_{\Phi }$ by replacing ${\vec{\Theta }}^{*}$ with ${\vec{\Theta }}^{cERF}$.

### A.2. Proof of proposition 2

According to Haufe et al. (2013), for a linear discriminative model with parameters $\hat{\Theta }$, the unique equivalent generative model can be computed as follows:

(A2) $$A\propto \Sigma_{X}\hat{\Theta }$$

In a binary (**Y** = {1, −1}) least squares classification scenario, we have:

(A3) $$A\propto \Sigma_{X}\Sigma_{X}^{-1}X^{T}Y=X^{T}Y=\mu^{+}-\mu^{-}$$

where Σ_**X**_ represents the covariance of the input matrix **X**, and μ^+^ and μ^−^ are the means of positive and negative samples, respectively. Therefore, the equivalent generative model for the above classification problem can be derived by computing the difference between the mean of samples in two classes that is equivalent to the definition of cERF in time-domain MEG data.

### A.3. The distribution of cosine similarity

The aim of this section is to illustrate that the probability density function (PDF) of the cosine similarity between two randomly drawn vectors in the high dimensional space (large *p*) is very close to normal distribution with zero mean and small variance. To do this, we first need to find the distribution of dot product in the uniform unit hyper-sphere. Let *a* and *b* be two uniformly drawn random vectors from a unit hyper-sphere in ℝ^*p*^. Assuming that γ is the angle between *a* and *b*, the distribution of cosine similarity is equivalent to the dot product < *a*.*b* >. Without loss of generality, let *b* be along the positive x-axis in the coordinate system. Thus, the dot product < *a*.*b* > is the projection of *a* on the x-axis, i.e., *x* coordinate of *a*. Therefore, for a certain value of γ, the dot product is a *p* − 1 dimensional hyper-sphere that is orthogonal to the x-axis (the red circle in Figure A2) and the PDF of the dot product is the surface area of *p* dimensional hyper-sphere constructed by the dot products for different γ values (the dashed blue sphere in Figure A2). To compute the area of this hyper-sphere we take the sum of the surface area of the *p* dimensional conical frustums over small intervals *dx* (the gray area in Figure A2):

(A4) $$\begin{array}{l} Pr(-1\leq x\leq 1)=2^{p-2}\pi \int_{-1}^{1}{\left(1-x^{2}\right)}^{p-2}\frac{dx}{1-x^{2}}\\ \;=2^{p-2}\pi \int_{-1}^{1}{\left(1-x^{2}\right)}^{p-3}dx \end{array}$$

where (1 − *x*^2^)^*p*−2^ is the surface area of the base of the cone (e.g., the perimeter of the red circle in Figure A2) and $\frac{dx}{1-x^{2}}$ is the slope size. Setting $t=\frac{x+1}{2}$ we have:

(A5) $$Pr(0\leq t\leq 1)=4^{p-2}\pi \int_{0}^{1}t^{\frac{p-3}{2}}{\left(1-t\right)}^{\frac{p-3}{2}}dt$$

which is a Beta distribution, where $\alpha =\beta =\frac{p-1}{2}$, that is a symmetric and unimodal distribution with mean 0.5. Because the PDF of *x* = 2*t* − 1 can be computed using a linear transformation of the above density function, it can be shown that the distribution of the dot product in unit hyper-sphere, i.e., the cosine similarity, is also a symmetric and unimodal distribution with 0 mean. Based on asymptotic assumption of Spruill (2007), for a large values of *p* this distribution converges to a normal distribution with $\sigma^{2}=\frac{1}{p}$. Therefore assuming large *p*, the distribution of cosine similarity for uniformly random vectors drawn from p-dimensional unit hyper-sphere is approximately $N\left(0,\sqrt{\frac{1}{p}}\right)$.

### A.4. Computing the bias and variance in binary classification

Here, using the out-of-bag (OOB) technique, and based on procedures proposed by Domingos (2000) and Valentini and Dietterich (2004), we compute the expected prediction error (EPE) for a linear binary classifier Φ under bootstrap perturbation of the training set. Let *m* be the number of perturbed training sets resulting from partitioning *S* = (*X*, *Y*) into *S*_*tr*_ = (*X*_*tr*_, *Y*_*tr*_) and *S*_*vl*_ = (*X*_*vl*_, *Y*_*vl*_), i.e., training and validation sets. If ${\hat{\Phi }}^{j}$ is the linear classifier estimated from the *j*th perturbed training set, then the main prediction $\Phi^{\mu }\left({\text{x}}_{i}\right)$ for each sample in the dataset can be computed as follows:

(A6) $$\Phi^{\mu }(x_{i})=\{\begin{array}{cc} 1 & if\;\frac{1}{k_{i}}\sum_{j=1}^{k_{i}}{\hat{\Phi }}^{j}(x_{i})\geq \frac{1}{2}\\ 0 & otherwise \end{array}$$

where *k*_*i*_ is the number of times that *x*_*i*_ is present in the test set^6^.

The computation of bias is challenging because the optimal model Φ^*^ is unknown. According to Tibshirani (1996a), misclassification error is one of the loss measures that satisfies a Pythagorean-type equality, and:

(A7) $$\begin{array}{l} \frac{1}{n}\sum_{i=1}^{n}ℒ(\Phi^{\mu }(x_{i}),\Phi^{*}(x_{i}))=\frac{1}{n}\sum_{i=1}^{n}ℒ(y_{i},\Phi^{\mu }(x_{i}))\\ \;-\frac{1}{n}\sum_{i=1}^{n}ℒ(y_{i},\Phi^{*}(x_{i})) \end{array}$$

Because all terms of the above equation are positive, the mean loss between the main prediction and the actual labels can be considered as an upper-bound for the bias:

(A8) $$\frac{1}{n}\sum_{i=1}^{n}ℒ(\Phi^{\mu }(x_{i}),\Phi^{*}(x_{i}))\leq \frac{1}{n}\sum_{i=1}^{n}ℒ(y_{i},\Phi^{\mu }(x_{i}))$$

Therefore, a pessimistic approximation of bias *B*(**x**_*i*_) can be calculated as follows:

(A9) $$B(x_{i})=\{\begin{array}{cc} 0 & if\;\Phi^{\mu }(x_{i})=y_{i}\\ 1 & otherwise \end{array}$$

Then, the unbiased and biased variances (see Domingos, 2000 for definitions) in each training set can be calculated by:

(A10) $$V_{u}^{j}(x_{i})=\{\begin{array}{cc} 1 & if\;B(x_{i})=0\;and\;\Phi^{\mu }(x_{i})\neq {\hat{\Phi }}^{j}(x_{i})\\ 0 & otherwise \end{array}$$

(A11) $$V_{b}^{j}(x_{i})=\{\begin{array}{cc} 1 & if\;B(x_{i})=1\;and\;\Phi^{\mu }(x_{i})\neq {\hat{\Phi }}^{j}(x_{i})\\ 0 & otherwise \end{array}$$

Then, the expected prediction error of Φ can be computed as follows (ignoring the irreducible error):

(A12) $$\begin{array}{l} \;EPE_{\Phi }(X)=\underset{Bias}{\underset{︸}{\frac{1}{n}\sum_{i=1}^{n}B(x_{i})}}+\\ \underset{Variance}{\underset{︸}{\frac{1}{nm}\sum_{j=1}^{m}\overset{n}{\underset{i=1}{\sum }}[V_{u}^{j}(x_{i})-V_{b}^{j}(x_{i})]}} \end{array}$$
