# Supplementary material for: Interpretability of Multivariate Brain Maps in Linear Brain Decoding: Definition, and Heuristic Quantification in Multivariate Analysis of MEG Time-Locked Effects
Source: Front Neurosci. 2017 Jan 23;10:619. doi: 10.3389/fnins.2016.00619 (PMC5253369; doi:10.3389/fnins.2016.00619)
Supplement: Supplementary file 1 [file Presentation1.PDF]

Supplementary Material: Interpretability of Multivariate Brain Maps in Linear Brain Decoding: Definition, and Heuristic Quantification in Multivariate Analysis of MEG Time-Locked Effects

Seyed Mostafa Kia, Sandro Vega-Pons, Nathan Weisz, and Andrea Passerini
*Correspondence:
Seyed Mostafa Kia
seyedmostafa.kia@unitn.it

This text provides supplementary materials and experiments for the main study with the title "Interpretability of Multivariate Brain Maps in Linear Brain Decoding: Definition, and Heuristic Quantification in Multivariate Analysis of MEG Time-Locked Effects".

1 INTERPRETABILITY IN THE LITERATURE: A QUICK META-ANALYSIS

The main purpose of this analysis is to illustrate the extensive usage of the term "interpretation" and its derivatives in the literature. This meta-analysis is limited to the bibliography of the main paper as a small sample of related studies. The books are excluded from our samples and the analysis is performed only on the journal and conference papers. The portable document format (PDF) file of the cited papers are converted to the text format, then the reference section of the papers are removed to avoid spurious results. Few papers are excluded from the analysis due to the problem in the conversion process and at the end 101 papers are used in our experiment. The AntConc [1] software is used for corpus analysis. Considering "interpretability", "interpretable", and "interpretation" as target words, three experiments are conducted:

1. In the first experiment the occurrence frequency of target words are computed in the corpus. Total number of 598 hits are reported.
2. In the second experiment the co-occurrence frequency of target words with "model", "classification", "parameter", "decoding", "method", "feature", and "pattern" are counted. In order to assess the local co-occurrence, the co-occurrence window is defined from 10 words before to 10 words after the target words. Figure S1(**A**) summarizes the result.
3. In the third experiment the co-occurrence frequency of target words with "reproducibility", "stability", "sparsity", and "plausibility" are counted. In this case since we were interested in the global frequency of co-occurrence, the co-occurrences window is defined from 100 words before to 100 words after the target words. Figure S1(**B**) summarizes the result.

Having in mind the limitations of our meta-analysis, e.g., small sample size and the bias in sample selection, we avoid to draw any conclusion out of these observations.

[1] Anthony, L. (2014). AntConc (Version 3.4.3) [Computer Software]. Tokyo, Japan: Waseda University. Available from http://www.laurenceanthony.net/

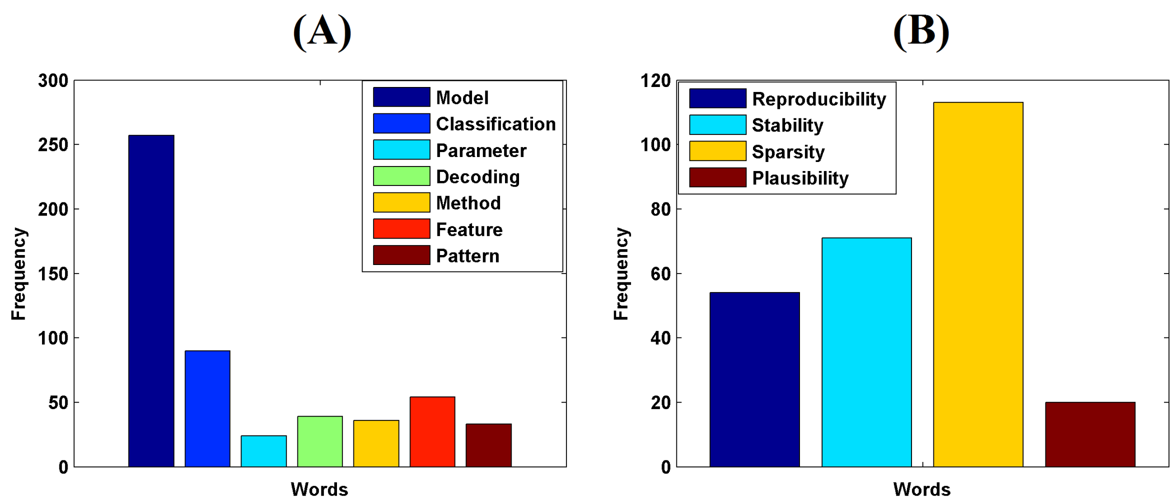

Figure S1. Frequency of co-occurrence of brain decoding related terms with "interpretation" and its derivatives.

2 USING ELASTIC-NET FOR CLASSIFICATION OF MEG DATA

As a complementary experiment, we repeated the experiment on the real MEG data using an elastic-net classifier. Elastic-net combines ℓ_1 and ℓ_2 penalization methods. Thus it has two hyper-parameters, λ and α, to control the amount of regularization, and the weights on the types of penalization, respectively. We have:

$$\hat{\Theta} = \underset{\Theta}{\operatorname{argmin}} \mathcal{L}(\mathbf{X}\Theta, \mathbf{Y}) + \lambda[\alpha \left\|\Theta\right\|_1 + (1-\alpha) \left\|\Theta\right\|_2^2] \tag{S1}$$

where $\|.\|_1$ and $\|.\|_2$ represent ℓ_1-norm and ℓ_2-norm, respectively. Therefore, the aim of the model selection is to find the best value for both λ and α. Here, we try to find the best hyper-parameter values among $\lambda = \{0.001, 0.01, 0.1, 1, 10, 50, 100, 250, 500, 1000\}$ and $\alpha = \{0, 0.0001, 0.001, 0.01, 0.1, 0.25, 0.5, 0.75, 0.9, 1\}$.

Figure S2 summarizes the mean and standard-deviation of the performance and interpretability of elastic-net across 16 subjects for different levels of regularization and sparsity. The results illustrate that increasing the amount of sparsity, by increasing α, increases the chance of performance-interpretability dilemma. While for a ridge model, with $\alpha = 0$, the performance and interpretability are consistent, by increasing the sparsity they show a divergent behavior. This observation illustrates the smooth, rather sparse, nature of the underlying effect in space and time.

These results are further analyzed in Figure S3 where $\hat{\Phi}_i^{\delta}$ and $\hat{\Phi}_i^{\zeta}$ are compared subject-wise in terms of their performance and interpretability. Similar to the Lasso model in the main text, the comparison shows that adopting ζ_Φ instead of δ_Φ as the criterion for model selection yields higher interpretable models by compensating a negligible degree of performance across all subjects. Figure S3(**A**) shows that employing δ_Φ provides on average slightly higher accurate models across subjects (0.83 ± 0.05) than using ζ_Φ (0.79 ± 0.04). On the other side, Figure S3(**B**) shows that employing ζ_Φ and compensating by 0.04 in the performance provides (on average) substantially higher level of interpretability across subjects

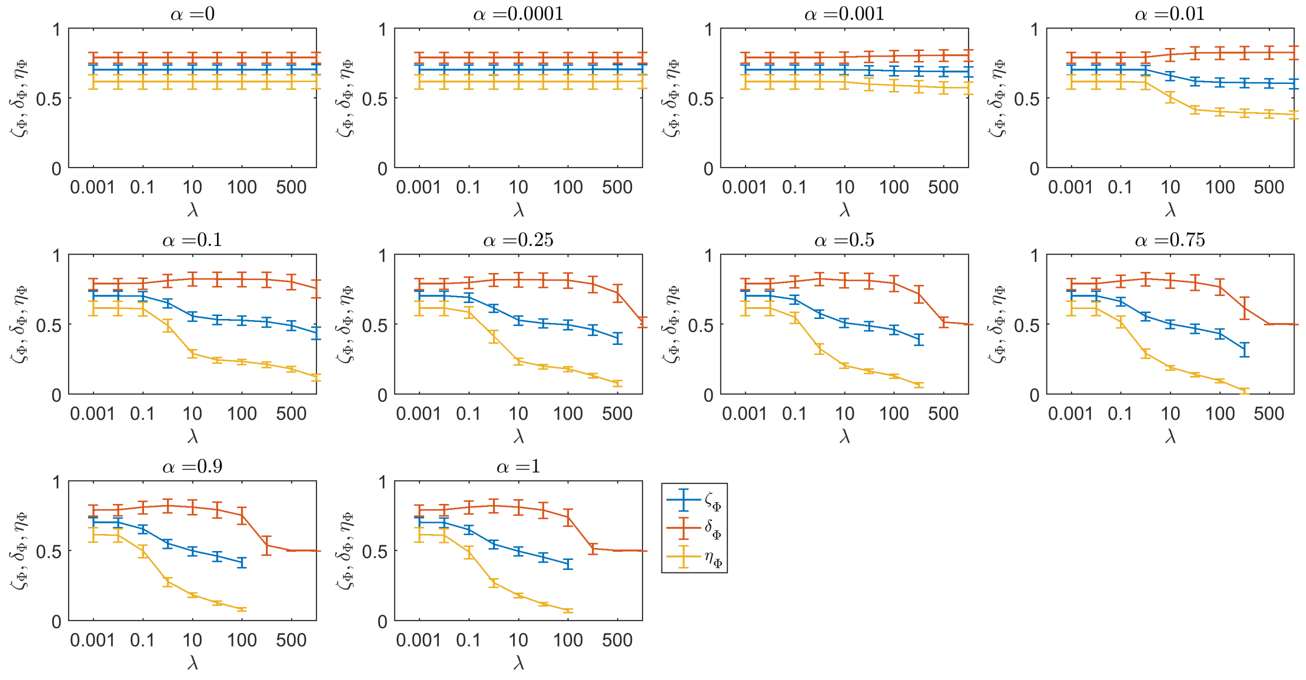

Figure S2. Mean and standard-deviation of the performance (δ_Φ), interpretability (η_Φ), and ζ_Φ of elastic-net model over 16 subjects. In this dataset, increasing the amount of sparsity increases the chance of performance-interpretability dilemma.

(0.62 ± 0.05) compared to δ_Φ (0.34 ± 0.11). The results obtained using elastic-net classifier are very similar to the ones of Lasso in the main text.

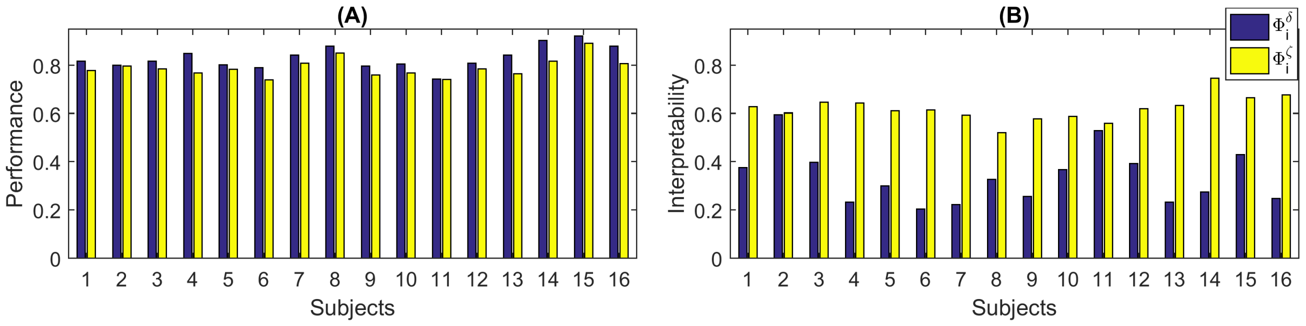

Figure S3. **(A)** Comparison between generalization performances of $\hat{\Phi}_i^\delta$ and $\hat{\Phi}_i^\zeta$ using elastic-net as classifier. **(B)** Comparison between interpretability of $\hat{\Phi}_i^\delta$ and $\hat{\Phi}_i^\zeta$ using elastic-net as classifier. The results obtained by elastic-net classifier are very similar to the Lasso model.
